# Adaptive immune responses to SARS-CoV-2 infection in severe versus mild individuals

**DOI:** 10.1038/s41392-020-00263-y

**Published:** 2020-08-14

**Authors:** Fan Zhang, Rui Gan, Ziqi Zhen, Xiaoli Hu, Xiang Li, Fengxia Zhou, Ying Liu, Chuangeng Chen, Shuangyu Xie, Bailing Zhang, Xiaoke Wu, Zhiwei Huang

**Affiliations:** 1 HIT Center for Life Sciences, School of Life Science and Technology, Harbin Institute of Technology, Harbin, 150080 China; 2 Department of Infectious Diseases, Heilongjiang Provincial Hospital, Harbin Institute of Technology, Harbin, 150030 China; 3 Harbin Blood Center, Harbin, 150056 China; 4 Centre for Reproductive Medicine, Heilongjiang Provincial Hospital, Harbin Institute of Technology, Harbin, 150030 China; 5 Department of Obstetrics and Gynecology, First Affiliated Hospital, Heilongjiang University of Chinese Medicine, Harbin, 150040 China

**Keywords:** Infectious diseases, Predictive medicine

## Abstract

The global Coronavirus disease 2019 (COVID-19) pandemic caused by SARS-CoV-2 has affected more than eight million people. There is an urgent need to investigate how the adaptive immunity is established in COVID-19 patients. In this study, we profiled adaptive immune cells of PBMCs from recovered COVID-19 patients with varying disease severity using single-cell RNA and TCR/BCR V(D)J sequencing. The sequencing data revealed SARS-CoV-2-specific shuffling of adaptive immune repertories and COVID-19-induced remodeling of peripheral lymphocytes. Characterization of variations in the peripheral T and B cells from the COVID-19 patients revealed a positive correlation of humoral immune response and T-cell immune memory with disease severity. Sequencing and functional data revealed SARS-CoV-2-specific T-cell immune memory in the convalescent COVID-19 patients. Furthermore, we also identified novel antigens that are responsive in the convalescent patients. Altogether, our study reveals adaptive immune repertories underlying pathogenesis and recovery in severe versus mild COVID-19 patients, providing valuable information for potential vaccine and therapeutic development against SARS-CoV-2 infection.

## Introduction

Coronavirus disease 2019 (COVID-19) caused by severe acute respiratory syndrome coronavirus 2 (SARS-CoV-2) has raised a global health emergency. Worldwide studies have contributed to the characterization, diagnosis, and treatment of the disease.^1–4^ However, the pathogenesis of SARS-CoV-2 infection in humans remains unclear. Previous studies on severe acute respiratory syndrome (SARS),^5^ Middle East respiratory syndrome,^6^ and influenza^7^ demonstrated that immune changes, especially those in peripheral blood lymphocyte subsets, play a critical role in defense against coronavirus infections. Consistently, several studies of COVID-19 patients showed that both humoral and cellular immunity are involved in the pathogenesis of COVID-19.^8–10^ Although most COVID-19 patients presented mild-to-moderate symptoms, some infected individuals did develop severe or critical outcomes. However, the immunological features associated with the disease severity remains largely unknown. In addition, earlier studies on the recovery of SARS patients have shown that complete restoration of peripheral lymphocyte may require a longer period.^11^ Thus, studies of the immune system of convalescent COVID-19 patients will facilitate understanding of their recovery state and establish the relationship between adaptive immune responses and disease severity if it exists.

Here, we collected peripheral blood CD3+ T cells and CD3−CD19+CD20+CD27+ antigen-experienced B cells (AEBCs) from five severe/critical and four mild-to-moderate convalescent COVID-19 patients. These cells were analyzed with single-cell TCR sequencing (scTCR-seq), single-cell BCR sequencing (scBCR-seq), and single-cell RNA sequencing (scRNA-seq). By using these techniques, we found SARS-CoV-2-specific shuffling of adaptive immune repertories and COVID-19-induced remodeling of peripheral lymphocytes. Characterization of variations in cell composition and functional status of the peripheral T and B cells of the recovered COVID-19 patients showed a more robust humoral immune response and T-cell immune memory in the severe patients (SPs). Data from scRNA-seq and scTCR-seq revealed concomitant responses of three major clusters of memory T cells in the adaptive immune system of the COVID-19 patients. Importantly, functional assays indicate that peptides derived from the M protein of SARS-CoV-2 are active in inducing T-cell response in most of the COVID-19 patients. Altogether, our data not only unveil the adaptive immunological features of convalescent COVID-19 patients but also provide information on the development vaccine and therapeutic agents against SARS-CoV-2 infection.

## Results

### Study design

**Fig. 1:**
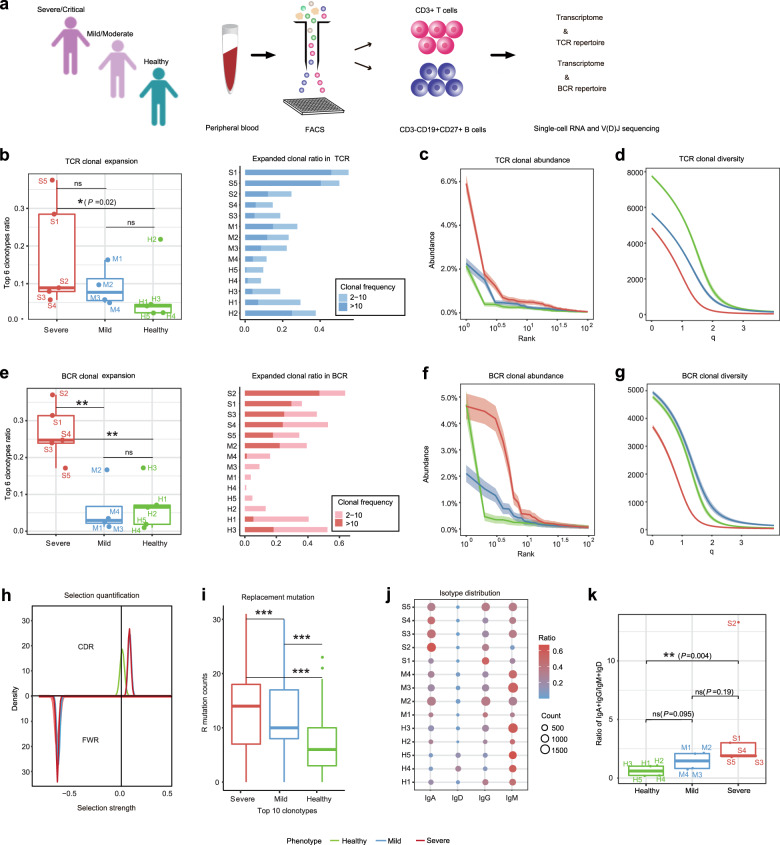
Characterization of T- and B-cell immune repertoires in COVID-19 convalescent patients. **a** Schematic representation of overall study design. Blood samples from recovered patients with severe/critical infection or mild/moderate infection, and healthy controls were collected and sorted to obtain CD3+ T cells and CD19+CD20+CD27+ antigen-experienced B cells (AEBCs). Sorted cells underwent high-throughput single-cell RNA and TCR/BCR V(D)J sequencing for further analyses. Characterization and comparison of TCR clonal expansion among severe/critical patients (SPs, red), mild/moderate patients (MPs, blue), and healthy controls (HCs, green), by quantifying the ratio of expanded clones (**b**), clonal abundance (**c**), and clonal diversity (**d**). Characterization and comparison of BCR clonal expansion among SPs, MPs, and HCs, by quantifying the ratio of expanded clones (**e**), clonal abundance (**f**), and clonal diversity (**g**). **h** Comparison of selection strength on complementary-determining region (CDR, top) and framework region (FWR, bottom) of the BCRs among SPs, MPs, and HCs by density plot. **i** Comparison of replacement mutation frequency on IGHV gene segments among SPs, MPs, and HCs by boxplot. **j** Comparison of isotype distributions among SPs, MPs, and HCs. **k** Comparison of the ratio of (IgA + IgG) to (IgD + IgM) in SPs, MPs, and HCs by boxplot. ***P < 0.001; **P < 0.01; *P < 0.05; n.s. not significant using Wilcoxon rank sum test for pairwise comparisons

To investigate whether immunological memory is established in the SARS-CoV-2-infected individuals, peripheral blood mononuclear cells (PBMCs) were isolated from a cohort of nine convalescent patients of COVID-19 and three age-matched healthy controls (HCs). These cells were further sorted into CD19+CD20+CD27+ AEBCs and CD3+ T-cell populations via flow cytometry. Listed in Supplementary Table 1 are the demographic and clinical characteristics of the nine COVID-19 donors including four mild-to-moderate cases (M1–M4), four severe cases (S1, S2, S3, and S5), and one critical case (S4). Diagnosis of SARS-CoV-2 was based on clinical symptoms, exposure history, and chest radiography, and SARS-CoV-2 infection was confirmed by using commercial quantitative PCR with reverse transcription assay on throat swab samples from the respiratory tract. The classification of disease severity for COVID-19 is based on the Chinese Clinical Guidance for COVID-19 Pneumonia Diagnosis and Treatment. The blood samples of these patients were collected about 4 weeks post discharge. Besides the three enrolled healthy donors (H1–H3), two more HCs were acquired from the Single Cell Immune Profiling Datasets of 10x genomics and labeled as H4 and H5. To understand how SARS-CoV-2 infection perturbs B-cell and T-cell immune repertoires, we applied scTCR-seq and scBCR-seq to sequence the V(D)J region of CD3+ T cells and AEBCs from 12 donors. After filtering, a total of 50,075 T cells (mean: 3070 cells for five SPs/critical patients, 3762 cells for four mild/moderate patients (MPs), 3936 for five HCs), and 29,899 AEBCs (mean: 2454 for SPs, 2652 for MPs, and 1404 for HCs) were obtained. Totally, 39,775 TCR and 21,339 BCR clones were acquired from these cells (Supplementary Table 2). Nonproductive (or nonfunctional) sequences were discarded in the following analysis. Furthermore, to further assess the cellular population composition and functional status of the peripheral T cells and AEBCs during the convalescing phase, we also conducted scRNA-seq on the T and AEBC cells from all the 12 enrolled donors. In total, 83,816 cells were recovered including 27,216 cells from SPs (mean: 5443 cells), 28,942 cells from MPs (mean: 7236 cells), and 27,658 cells from HCs (mean: 5532 cells) (Fig. 1a). By integrative analysis of single-cell transcriptome and immune profiling, we aim to reveal the adaptive immunological features related to the disease severity in the convalescent COVID-19 patients (Fig. 1a).

### T- and B-cell immune repertoires in COVID-19 patients

A direct consequence of SARS-CoV-2 infection is TCR or BCR clonal expansion and expanded memory B-cell clones have been used to screen neutralizing antibodies against SARS-CoV-2.^12^ We first investigated the clonal expansion by quantifying the ratio of expanded clones, clonal abundance distributions^13^, and clonal diversity^14^ in the whole TCR or BCR repertories. We observed comparable T-cell clonal expansion in the SPs and MPs (Fig. 1b), whereas overall increased TCR clonal abundance (Fig. 1c) and reduced diversity (Fig. 1d) were observed in the SPs. Notably, more expanded BCR clones (Fig. 1e), increased clonal abundance (Fig. 1f), and reduced clonal diversity (Fig. 1g) were present in the SPs compared with the MPs and the HCs. In contrast, no significant BCR clonal expansion was observed in most of the MPs (except for M2 (56 years), the oldest individual in the mild group (mean: 46 years)) (Fig. 1e). Taken together, these results show the persistence of robust T-cell responses in most of the convalescent COVID-19 patients, and stronger B-cell immune responses to SARS-CoV-2 infection in the SPs than in the MPs.

To further characterize BCR clonal expansion in these patients, we applied BASELINe^15^ to the entire Ig sequences to estimate the selection strength in the CDR and FWR regions. The analysis revealed a clear positive selection in the CDR region of the COVID-19 patients but not in the HCs (Fig. 1h), suggesting that the patients’ Ig repertoires were modified to produce high-affinity memory and plasma cells against SARS-CoV-2 infection. An increased frequency of replacement (non-synonymous) mutations occurs in the BCR CDR region during the process of antigen-driven BCR affinity maturation.^16^ Indeed, we observed higher mutation frequencies of the top expanded clones in the SPs and MPs compared with the HCs (Fig. 1i). Consistently, higher mutation frequencies were also found in the more expanded clones than in the less expanded clones (Supplementary Fig. 1). IgG and IgA isotypes were overrepresented in the convalescent patients, especially in the severe cases in comparison with the HCs (Fig. 1j). The ratio of (IgA + IgG) to (IgD + IgM) increased with disease severity in these COVID-19 patients (Fig. 1k), suggesting that SARS-CoV-2 induced a more intensive antibody response in the SPs than in the MPs.

**Fig. 2:**
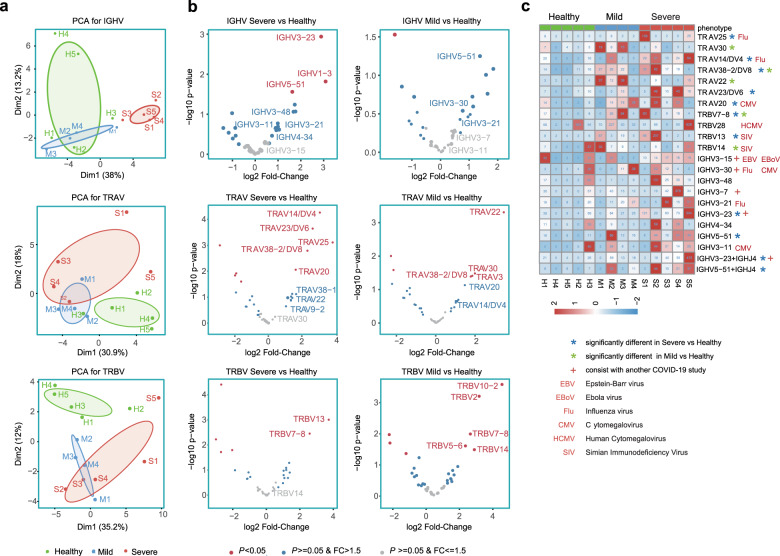
Comparison of V gene usage of IGH, TRA, and TRB among SPs, MPs, and HCs. **a** Principal component analysis (PCA) for IGHV-gene usage and TRAV or TRBV-gene usage in SPs (S1–S5), MPs (M1–M4), and HCs (H1–H5). **b** Volcano plot representing differences of IGHV-gene usage in BCR repertoires or TRAV/TRBV-gene usage of TCR repertoires. Positive fold-change values denote more frequent IGHV genes or TRAV/TRBV genes in COVID-19 patients. Genes with *P* value < 0.05 are displayed in red. Genes with a *P* value > 0.05 but fold-change (FC) value > 1.5 are displayed in blue. **c** Heatmap of reprehensive gene segments enriched in SPs or MPs compared with in HCs. Colors denote frequencies of each V gene segment used in each sample. The V genes overpresented in SPs or MPs (*P* value < 0.05) are indicated using a blue or a green star, respectively. Other known biasedly used gene segments related to virus-specific antibodies are marked using relevant virus name (EBV, EBoV, Flu, CMV, HCMV, and SIV). Genes consistent with another COVID-19 recovered patient study^10^ are denoted with a cross

Next, to reveal the unique gene patterns and preferences for BCR or TCR in the COVID-19 patients, we compared the usage of V(D)J genes among the participants. We first evaluated the overall immune repertoire differences among them. Principle component analysis (PCA) was used to explore the inter-individual variations based on the usage properties of IGHV genes of BCRs and TRAV or TRBV genes of TCRs. PCA plot showed that clonotype composition of the healthy and the two distinct severity groups of COVID-19 patients are apart from each other (Fig. 2a), suggesting shuffling of the V gene usage preference in the adaptive immune repertories of the patients. Importantly, the IGHV-gene repertoire was remarkably biased in the SPs (Fig. 2b, c), with IGHV3 family genes including *IGHV3-23* (16.8%), *IGHV3-7* (10.2%), *IGHV3-48* (4.5%), *IGHV3-21* (3.3%), *IGHV3-11* (2.24%), and *IGHV3-15* (1.64%) accounting for ~40% of the whole expanded cohort. Similarly, enhanced usage of the IGHV3 family genes was also observed in human antibodies against other viruses such as cytomegalovirus (CMV),^17^ influenza virus,^18^ and Ebola virus.^19^ Interestingly, IGHV4-34 B-cell clones, rarely present in IgG memory B cells from healthy individuals,^20^ were highly represented in one of the severe cases (S2) (Fig. 2c). Moreover, the top two pairing VJ segments *IGHV3-23–IGHJ4* and *IGHV5-51–IGHJ4* of BCR clones in the SPs appeared SARS-CoV-2 specific^10^ (Fig. 2c). When comparing severe group with the mild group, IGHV3-23, IGHV3-48, IGHV1-2, and IGHV4-34 were dominated in severe group (Supplementary Fig. 2). We similarly discerned gene usage preference of *TRAV14/DV4*, *TRAV20*, *TRAV23/DV6*, *TRAV25*, *TRBV13*, *TRBV14*, and *TRBV28* in the SPs and MPs (Fig. 2a–c), and some of them (*TRAV14/DV4*, *TRAV20*, and *TRBV13*) were also observed to expand in response to a range of viruses including influenza virus,^21^ CMV,^22^ and simian immunodeficiency virus (SIV).^23^

To identify convergent antibodies for COVID-19, we pooled the BCR data from the 14 individuals together and carried out clonal grouping using Change-O toolkit,^24^ based on common genes of IGHV and IGHJ and nucleotide similarity of CDR3 sequences. Public antibody sequences present in more than a single donor were identified and extracted for multiple alignment analysis of their CDR3 regions (Supplementary Fig. 3). The data from the analysis revealed a repertoire of public clusters (0.786, 0.6, and 0.92% of total IgA, IgG, and IgM clusters) in the nine COVID-19 patients but not in the five HCs (0.156, 0, and 0% of total IgA, IgG, and IgM clusters) (Supplementary Fig. 4a), presumably due to the infection of SARS-CoV-2.^25^ In total, we identified 19 convergent IgG and 25 IgA antibodies shared by the COVID-19 patients (Supplementary Table 3, the human antibodies sequences will be provided upon request), though their SARS-CoV-2 neutralizing activity warrants future investigations. During preparation of the manuscript, a recently published paper reported^26^ convergent antibodies of *IGHV3-30*/*IGKV1-39* from two COVID-19 convalescent donors. Next, we used GLIPH^27^ to analyze TCRβ sequences and grouped them according to the CDR3 sequence similarity. Likewise, we found more public TCR clusters in the SPs than in the MPs or HCs (1.8, 0.62, and 0.66% of total TCR clusters in SPs, MPs, and HCs, respectively) (Supplementary Fig. 4b). Taken together, these results support the notion that severe and mild COVID-19 patients experience distinct humoral and cell-mediated adaptive immune responses.

### Characterization of variations in cell composition and functional status of the peripheral T and B cells in recovered COVID-19 patients

**Fig. 3:**
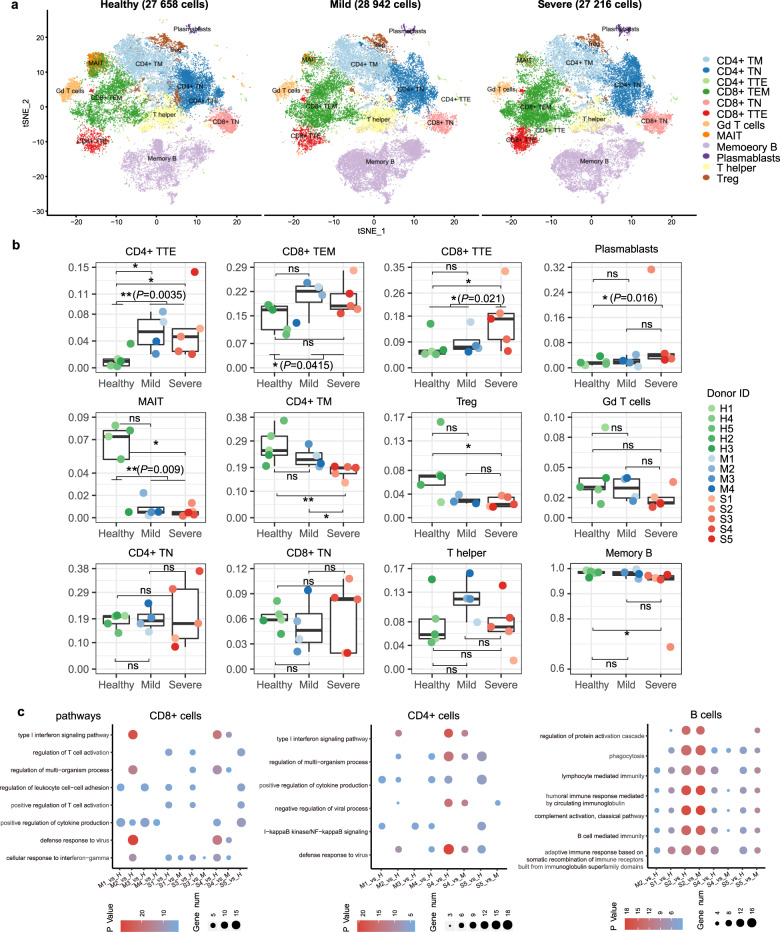
Characterization of variations in cell composition and functional status of the peripheral T and B cells of the recovered COVID-19 patients. **a** Two-dimensional t-SNE visualization of 12 major cell types identified from healthy controls (HCs, left), mild patients (MPs, middle), and severe patients (SPs, right) including four for CD8+ T cells, five for CD4+ T cells, one for γδ T cells, and two for B cells. Each dot corresponds to one single cell, and colored according to cell types. The number of total cells showed in each t-SNE map was labeled on the top. **b** Boxplot representing proportions of each cell type in each sample. Each dot corresponds to one sample, and colored by group-specific color scheme (severe, red gradients; mild, blue gradients; healthy, green gradients). **P < 0.01; *P < 0.05; n.s. not significant by Wilcoxon rank sum test. **c** Dot plot depicting upregulated canonical pathways in CD4+ T cells, CD8+ T cells, and AEBCs shared by multiple COVID-19 patients. Differentially expressed genes and their enriched pathways were calculated as each COVID-19 patient relative to all healthy controls (S# versus H or M# versus H), and each severe patient relative to all mild cases (S# versus M)

To characterize the adaptive immune system of the convalescent COVID-19 patients and understand their recovery state, we performed scRNA-seq analysis on CD3+ T cells and AEBCs from the SPs (S1–S5), MPs (M1–M4), and HCs (H1–H5) using Cell Ranger count pipeline. After quality control, a total of 83,817 cells were obtained for downstream analysis. Using a Louvain clustering algorithm^28^ and automated reference-based annotation tools (Scibet^29^ and SingleR^30^) combined with expression of canonical genes, we identified ten distinct clusters representing different T-cell subsets and two distinct clusters representing different B-cell subsets (Supplementary Table 4 and Supplementary Fig. 5). Then t-distributed stochastic neighbor embedding (t-SNE) was performed to visualize the cells in 2D space (Fig. 3a). Mucosal associated invariant T (MAIT) cells were characterized by the invariant alpha chain *TRAV1-2* in conjunction with *TRAJ33*, *TRAJ20*, or *TRAJ12*. Vγ9/Vδ2+ T cells (the major Gd T-cell fraction in the peripheral blood) and cytotoxic T cells were identified in the T-cell subsets based on expression of the marker genes *TRDV2*, *TRGV9*, and *TRDC* and cytotoxic effector molecules of *GZMA*, *GZMB*, *PRF1*, and *NKG7*, respectively (Supplementary Fig. 5).

To reveal COVID-19-driving changes in the compositions of the T- or B-cell subsets, we quantified the proportions of all cell types in each individual from the SP, MP, and HC categories (Fig. 3b). Overall, we observed no significant difference in the CD4/CD8 naive, T helper, and Gd T-cell subsets among the SPs, MPs, and HCs. In contrast, both SPs and MPs exhibited elevated percentages of CD8+ effector memory (TEM) cells (mean: ~20.1%) compared with HCs (mean: ~14.6%, *P* = 0.0415, Wilcoxon rank sum test), suggesting that the proliferation of these cells correlates with COVID-19. Notably, the subset of CD8+ TEM cells re-expressing *CD45RA* (named CD8+ terminal effector (TTE) cells) were significantly higher in the SPs (mean: ~17%) than those in the MPs and HCs (mean: ~8%, *P* = 0.021, Wilcoxon rank sum test). These CD8+ TTE cells are mostly cytotoxic T cells expressing cytotoxic effector genes, such as *GZMA*, *GZMB*, *PRF1*, and *NKG7*. Indeed, we found that the COVID-19 patients possessed a small CD4+ TTE cell population (mean: ~5.6%), which was barely detected in the healthy individuals (mean: ~1.3%, *P* = 0.0035, Wilcoxon rank sum test). Notably, the MAIT cells were substantially diminished in both SPs and MPs (patient versus health, *P* = 0.009, Wilcoxon rank sum test) compared with the HCs. Although the significance of this is still unclear, this result indicates that the immune system of COVID-19 patients has not been fully restored at early recovery stage, consistent with the study of recovery of SARS patients. Of note, depletion of MAIT cells was also observed in other viral diseases, such as HIV and HCV.^31^ In contrast, SARS-CoV-2-induced depletion of Vγ9/Vδ2+ T cells^8^ was recovered in the convalescent patients (Fig. 3b). In addition, plasmablasts were enriched in the SPs (*P* = 0.016), further supporting the conclusion that a more robust humoral immune response is stimulated in severe cases compared with the mild cases (Fig. 3b).^4,32^

To further characterize the functional status of T cells and AEBCs in the convalescent SPs and MPs, we conducted differentially expressed gene (DEG) analysis, gene pathway enrichment analysis, and gene set enrichment analysis across the CD4+, CD8+, and memory B-cell subsets. Considering the highly heterogeneous immune responses among the COVID-19 patients,^4^ we detected differentially expressed immune-related and inflammation-related pathways in every patient compared with HCs in CD4+ T cells, CD8+ T cells, and AEBCs, respectively, and further investigated the upregulated canonical pathways shared by multiple COVID-19 patients (Fig. 3c). As a result, we found that the signaling pathways of “cytokine production” and “leukocyte cell-cell adhesion” are the most commonly upregulated in either the CD4+ or the CD8+ T cell sets of the COVID-19 patients (Fig. 3c). The T-cell activation pathway was activated in the CD8+ T cells in three severe cases, whereas the pathways involving antiviral immunity, such as “type I interferon signaling” and “defense response to virus” were highly upregulated in the T cells of S4 and M2. Compared with the HCs and the MPs, the SPs clearly expressed more genes in the pathways of “B-cell-mediated immunity,” “complement activation,” “humoral immune response mediated by circulating immunoglobulin,” and “phagocytosis,” indicating a more extensive humoral immune response in the severe cases. This conclusion is further supported by analyses of BCR clonal expansion (Fig. 1e) and plasmablasts enrichment (Fig. 3b). The upregulation of the complement pathway in the SPs suggests a systemic pro-inflammatory response induced by SARS-CoV-2 infection as documented before.^33^ Severe COVID-19 patients are normally associated with increased levels of interleukin *(IL)*-6, *IL*-10, and tumor necrosis factor*-α*.^34,35^ However, substantial expression of pro-inflammatory cytokines such as IL-6, *IL*-1*ß*, *IL*-2, *IL*-10, *IL*1*A*, *IL*-8, and *CXCL*-10 was not detected in most of the profiled T or B cells from all COVID-19 samples (Supplementary Fig. 6), suggesting that *(IL)*-6, *IL*-10, and probably other cytokines returned back to normal levels after recovery of these COVID-19 patients. Notably, the mitogen-activated protein kinase (MAPK) pathway (i.e., *FOS*, *JUN*, *JUNB*, and *DUSP1*) was greatly suppressed in all recovered patients compared with that in the HCs. This is in full agreement with a previous study suggesting that inhibition of the MAPK signaling pathway is a recovery sign of COVID-19 patient (Supplementary Fig. 7).^36^

### Generation of specific T-cell subsets in response to SARS-CoV-2

**Fig. 4:**
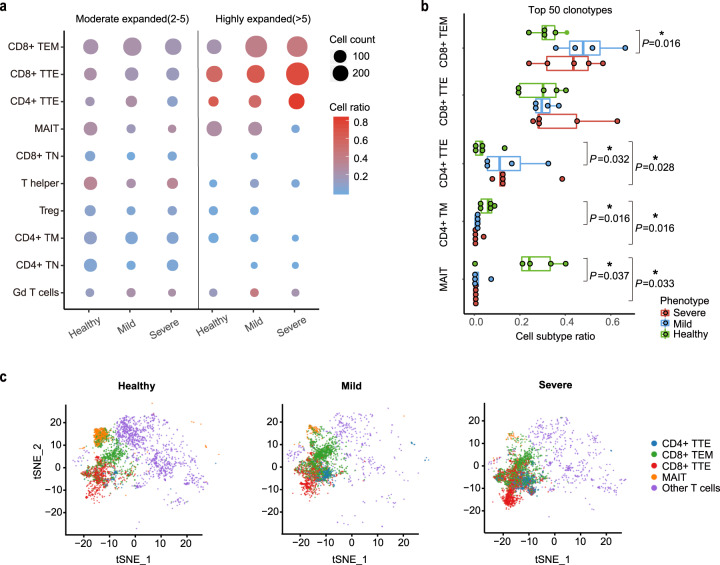
Comparison of TCR clonal expansion in T-cell subsets among severe patients (SPs), mild patients (MPs) and healthy controls (HCs). **a** Dot plot depicting clonal expansion status of distinct T-cell subsets in HCs, MPs, and SPs. Dot size denotes cell counts in each T-cell subset belonging to more expanded (frequency > 5) or less expanded (2 ≤ frequency ≤ 5) clonotypes. Red-blue color range denotes the ratio of expanded cells occupied in each T-cell subset. **b** Boxplot depicting the top 50 TCR clonotypes in each donor consists of various T-cell subsets of different proportions. *P < 0.05; n.s. not significant using Wilcoxon rank sum test for pairwise comparisons. **c** T-SNE projection of clonally expanded T cells (blue, CD4+ TTE; green, CD8+ TEM; red, CD8+ TTE; orange, MAIT; violet, other T cells) in HCs, MPs, and SPs

To identify SARS-CoV-2-specific T-cell subsets in the convalescent COVID-19 patients, we combined scRNA-seq and scTCR-seq to assess clonal expansion of different T-cell subsets. Full-length TCRs with both alpha and beta chains were obtained from 15,818 T cells of the 10 clusters in COVID-19 patients, including 11,805 harboring unique TCRs and 4013 (25.4%) harboring repeated TCRs, indicative of clonal expansion of T cells in COVID-19 patients. Compared with other T-cell subsets, CD8+ TEM, CD8+ TTE, and CD4+ TTE were substantially clonally expanded in the COVID-19 patients, with more expanded clonotypes containing more CD8+ TEM and CD8+/CD4+ TTE (Fig. 4a and Supplementary Fig. 8). The top 50 TCR clonotypes comprised, on average, 48.67% CD8+ TEM or 75.52% CD8+ TTE cells or 75.76% CD4+ TTE cells in the SPs, whereas a lower copy number of these clones existed in the HCs (Fig. 4b). These clonally expanded T cells may represent SARS-CoV-2-specific CD8+ or CD4+ T cells. Interestingly, the clonally expanded T cells showed an aggregative distribution on the t-SNE map (Fig. 4c), indicating transcriptional homogeneity in the COVID-19 patients and further supporting the idea that they are SARS-CoV-2 specific. Altogether, our data indicate concomitant response of CD8+ and CD4+ T cells in the adaptive immune system of the COVID-19 patients, and the clonally expanded CD8+ effector cells in the patients may ultimately develop into long-lived memory T cells. These data are further supported by a recent study showing a robust adaptive immune response in mild COIVD-19 patients.^3^ Interestingly, MAIT clones were not expanded in the COVID-19 patients but were preferentially amplified in the healthy donors, though the significance of this remains unclear.

**Fig. 5:**
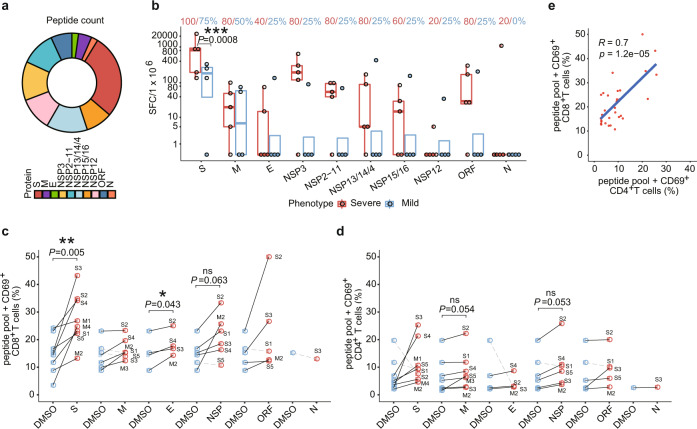
Assessment of SARS-CoV-2 specific T-cell immunity in COVID-19 convalescent patients by IFN-γ ELISpot and FACS analysis. **a** Composition and fraction of peptides used in ten SARS-CoV-2 antigen pools. **b** Boxplot comparing the number of spot-forming cells (*y* axis) from ELISpot assay between stimulated severe and mild samples for each antigen group (*x* axis). Each donor was tested in duplicates. For each donor, the average number of spots from stimulated samples subtract that of the unstimulated samples was shown as a dot on the plot. Negative values were set to zero. Red and blue dots indicate SPs and MPs, respectively. ***P < 0.001 by unpaired Student’s *t* test. SFC spot-forming cells. The blue or red text on top indicates the percentage of stimulated mild or severe donors shown positive in ELISPOT, respectively. Antigen-specific CD69+CD8+ T cell (**c**) and CD4+ T cell (**d**) reactivity in ELISpot-positive COVID-19 cases between the negative control (DMSO, blue dots) and antigen pool stimulations (red dots). **P < 0.01; *P < 0.05 by paired *t*-test. **e** Pearson’s correlation between SARS-CoV-2-specific CD4+ T cells (*x* axis) and SARS-CoV-2-specific CD8+ T cells (*y* axis)

To experimentally validate the SARS-CoV-2 specific T-cell immunity in the COVID-19 convalescent patients, we synthesized 276 potential T-cell epitope peptides from all the 29 proteins of SARS-CoV-2,^37–39^ with 210 corresponding to HLA class I CD8+ T-cell epitopes and 66 to HLA class II CD4+ T-cell epitopes (Fig. 5a). These peptides were divided into ten groups (as described in Supplementary Table 5) to stimulate PBMCs followed by interferon gamma (IFN-γ) ELISpot analysis. IFN-γ-secreting T cells from each donor were detected and counted. A donor was considered positive to a peptide group when there was a more than twofold increase in the numbers of IFN-γ-secreting T cells in the peptide-treated sample compared with the unstimulated control. At this cutoff point, we found that all recovered patients demonstrated specific memory T-cell responses against at least one group of the SARS-CoV-2 peptides (Fig. 5b). IFN-γ-secreting T cells in response to the S and the M peptides were detected in all SPs and most MPs (75 and 50%). Interestingly, the S peptides stimulated a markedly higher percentage of IFN-γ-secreting cells in both SPs and MPs than the other types of viral peptide. Each of the SPs demonstrated T-cell responses (mean: 12-fold increase) against at least five viral proteins, whereas most of the MPs (75%) only responded to the S protein (mean: 3.46-fold). Taken together, the ELISPOT data indicate that the memory T cells specific for the S and other proteins were generated and sustained in the COVID-19 convalescent patients, which is further supported by a recent study.^40^ Importantly, we found that that the SPs exhibited stronger T-cell immune responses than the MPs, suggesting that SPs may retain a more robust T-cell memory against SARS-CoV-2 than MPs.

To further determine the subpopulations of cytokine-producing memory T cells, totally 31 ELISPOT-positive PBMCs from recovered donors were stained with antibodies against CD4/CD69 and CD8/CD69 for FACS analysis. SARS-CoV-2 Spike-specific CD8+/CD69+ T cells were detected in all tested cases (*P* = 0.005 versus DMSO control, paired *t*-test, Fig. 5c). CD8+/CD69+ T cells were detected in 71, 75, 83, and 80% tested cases after stimulation with M, E, NSP, and ORFs (ORF3, ORF3a, ORF6, ORF7a, ORF7b, ORF8, and ORF10), respectively. These results show that the convalescent COVID-19 patients generated a substantial CD8+ memory T-cell response against SARS-CoV-2 antigens. CD4+ T-cell activation in response to S, M, E, NSP, and other ORFs was also detected in most tested cases (≥75%) (Fig. 5d), which is well correlated with CD8+ T responses (*R* = 0.7, *P* = 1.2e−05, Pearson’s correlation test, Fig. 5e).

## Discussion

Differential adaptive immune responses between severe and mild COVID-19 patients remain largely unknown. This study aims to investigate the dynamics of antibody levels and T-cell responses against SARS-CoV-2 according to disease severity. In this respect, we observed multiple differences between the SPs and the MPs including TCR and BCR clonal expansion and diversity (Fig. 1), isotype distribution of antibody sequences (Fig. 1), V(D)J gene segments usage preference (Fig. 2), and dysregulation of peripheral blood lymphocyte subsets (Fig. 3). Higher levels of BCR clonal expansion (Fig. 1) and B-cell activation (Fig. 3) are present in the SP group, indicating a more robust humoral immune response happened in severe infection. Taken together, these results denote that SPs and MPs may experience different cellular and humoral immune responses, which likely are related to different degrees of disease severity.

The upregulated immune pathways indicate that the immune systems of the convalescent patients still remain active, although they are no longer symptomatic. This agrees well with the notion that a longer period of time may require for complete restoration from the SARS-CoV-2 infection. A detailed analysis of peripheral immune cell compositions and functional status of peripheral lymphocyte is essential to comprehensively evaluate patient recovery stage.

Early studies have demonstrated an important role of type I and III IFN signaling pathways in SARS-CoV-2 or SARS-CoV infection,^4,41,42^ and proposed prophylactic and therapeutic potential of these interferons in COVID-19 patients. Our data show that T-cell IFN response could only be detected in one critical infection case (S4) and one mild case (M2) (Fig. 3c and Supplementary Fig. 6). This may reflect the heterogeneous response of IFN induction in COVID-19 patients^4^ and even their heterogeneous pathogeneses. However, activation of IFN-mediated pathways of T cells during SARS-CoV-2 infection and its relationship with clinical outcomes and antiviral immunity remains to be elucidated. Interestingly, M2 is the only individual in mild group that shares some common features with severe COVID-19 patients in single-cell immune profiling and transcriptome, such as highly clonal expansion (Fig. 1b, e) and upregulated pathways (Fig. 3c). M2 is also the only enrolled COVID-19 patient with hypertension and diabetes. This may suggest that some chronic basic diseases may associate with COVID-19^43^ via affecting adaptive immune responses to SARS-COV-2. The heterogeneity in immune responses of COVID-19 patients was also observed in the youngest (44 years) patient S2. The IGHV4-34 B-cell clones, rarely present in IgG memory B cells from healthy individuals,^20^ were highly expressed in this patients. Interestingly, a TCR clone (frequency = 38) from the CD8+ effector T cells of S2 was predicted to target an antigen peptide (LLLGIGILV) from an interferon-stimulated gene and human bone marrow stromal cell antigen 2 (*BST2*) (Supplementary Fig. 9). This suggests the possibility that autoimmunity induced by SARS-CoV-2 infection may occur to S2, thus contributing to the severe symptom of the patient.

To meet the urgent need for vaccine development, it is imperative to understand the magnitude and composition of human CD4+ and CD8+ T-cell responses to SARS-CoV-2. Our data of scRNA-seq and scTCR-seq revealed generation of specific T-cell subsets induced by the infectious virus. In strong support of this conclusion, many peptides derived from SARS-CoV-2 induced specific memory T-cell responses in the recovered patients. Interestingly, in addition to peptides from the S protein, which acts as the major target in most vaccine development efforts,^40^ peptides derived from the M protein are also reactive in T-cell response in most of the COVID-19 patients. Our data showed that the reactive major viral proteins are potential targets for future vaccine development. Our data further revealed concomitant response of three major clusters of memory T cells (CD8+ TEM, CD8+ TTE, and CD4+ TTE) in the adaptive immune system of the COVID-19 patients. This appears to be independent of the severity of the patients and is consistent with the data that robust adaptive immune responses are activated in mild COIVD-19 patients.^3^

## Materials and methods

### Ethics statement

This study was conducted according to the principles expressed in the Declaration of Helsinki. Ethical approval was obtained from the Research Ethics Committee of Heilongjiang Provincial Hospital (2020-112). All participants provided written informed consent for sample collection and subsequent analyses.

### Patients and clinical sample collection

All nine patients with COVID-19 in this study were enrolled from the Heilongjiang provincial Hospital from March to April, 2020. The clinical classifications was defined as mild to moderate, severe, and critical, according to the “Diagnosis and Treatment Protocol of COVID-19 (the 7th Tentative Version)” by the National Health Commission of China issued on 3 March 2020 (http://www.nhc.gov.cn/yzygj/s7653p/202003/46c9294a7dfe4cef80dc7f5912eb1989.shtml).

Briefly, moderate cases have fever, respiratory symptoms, and pneumonia evidenced by computed tomography (CT) imaging. This study enrolled two patients with moderate infection with bilateral pneumonia, labeled M1 and M2, two patients with mild infection, labeled M3 and M4. Patients with severe pneumonia were diagnosed on the basis of one of the following criteria: (1) respiratory distress with respiratory rate ≥ 30 times min^−1^; (2) fingertip oxygen saturation ≤ 93% at room air; (3) ratio of partial pressure of arterial oxygen to fraction of inspired oxygen (PaO_2_/FiO_2_) ≤ 300 mmHg (1 mmHg = 0.133 kPa); and (4) obvious progression of lesions in 24–48 h shown by pulmonary CT imaging > 50%. Two patients with severe pneumonia were enrolled, labeled as S1, S2, S3, and S5. These four patients showed PaO_2_/FiO_2_ ≤ 300 mmHg. The patients with critical pneumonia (S4) were diagnosed on the basis of one of the following criteria: (1) respiratory failure and an artificial airway required for invasive mechanical ventilation; (2) shock; and (3) combined failure of other organs that required intensive care unit monitoring. S4 is the patient with critical pneumonia received invasive mechanical ventilation.

### Isolation of PBMCs

PBMCs were isolated from anticoagulant blood using Ficoll under the biosafety level 2 facility. To isolate PBMCs, blood was diluted with the same volume of PBS, then gently put on the equal volume of Ficoll in a 15 ml centrifuge tube and centrifuged for 35 min at 700 × *g* without brake. Discard the first layer that contains serum and platelet, carefully transfer the buffy coat to a new centrifuge tube, wash with 10 ml PBS, and centrifuge at 350 × *g*, room temperature, 5 min. The supernatant was discarded and the pellet was washed again. After that, recollect these PBMCs, count the cell number, and store in the liquid nitrogen.

### Isolation of T and B cells

Approximately 20 ml of human peripheral blood was obtained and placed on ice, and returned to room temperature before use. PBMCs isolation was processed within 2 h in a BSL-2 laboratory. PBMCs were isolated from whole blood samples by Ficoll density gradient centrifugation. Then, cells were counted in 0.4% Trypan blue, 1 × 10^7^ PBMCs was prepared for flow cytometry and cell sorting, and residual cells were centrifuged and resuspended at a concentration of 2 × 10^6^/ml for further use.

### Flow cytometry and cell sorting

A FACS Aria cell sorter (BD Biosciences) was used to isolate T cells (CD3+), and CD3−CD19+CD20+CD27+ AEBCs (comprised of mostly memory B cells with a small number of peripheral plasmablasts). Surface marker staining and cell sorting was performed by pelleting and resuspending cells in FACS buffer (2% FBS in PBS) with antibodies at indicated concentrations for 20 min at room temperature in the dark. Cells were washed once in the FACS buffer before resuspension. PBMCs were filtered with a 100-µm nylon cell strainer to remove clumps and debris before flow cytometry.

### IFN-γ ELISpot

IFN-γ-secreting T cells were detected by Human IFN-γ ELISpot pro 3 kits (MABTECH AB, Sweden) according to the manufacture’s protocol. Briefly, frozen PBMCs in cryotubes were placed in a 37 °C water bath till completely thawed, then transferred to a new 15-ml centrifuge tubes containing 10 ml complete RPMI medium (90% RPMI-1640, 10% heat-inactivated FBS, 1% penicillin–streptomycin), and centrifuged at 1000 rpm, room temperature for 5 min. The cell pellet was resuspended in 2–4 ml complete RPMI and balanced at 37 °C with 5% CO2 for 6 h. Cell concentrations were adjusted to ~2 × 10^6^/ml and plated at 100 K per well in duplicate and incubated for 36 h with 10 μg/ml of peptide pools (the concentration of each peptide is 10 μg/ml). Spots were then counted using an Elispot Reader System (AT-Spot2100, atyx). The number of spots was converted into the number of spots-forming cells (SFCs) per million cells and the mean of duplicate wells was plotted.

We collected 276 peptides identified as potential T-cell epitopes for SARS-CoV-2 by at least two previous studies.^37–39^ This pool of 9–15-mer peptides was derived from 29 proteins spanning the whole proteome of SARS-CoV-2, including 76 epitopes from spike (S), 6 from nucleocapsid (N), 12 from membrane (M), 6 from envelope (E), 157 from Orf1ab (Nsp1-Nsp16), and 20 from 7 other ORFs (ORF3, ORF3a, ORF6, ORF7a, ORF7b, ORF8, and ORF10). We divided these 276 peptides into ten groups, with peptides from the same protein in the same group. Details of the groups are shown in Supplementary Table 5.

### Cells activation and surface marker staining

Cells were thawed in complete RPIM 1640 culture media and then left overnight at 37 °C, 5% CO2. 2e5 PBMCs were plated in a 96-well plate and stimulated with peptides that shown positive in ELISPOT. After 24 h culture, PBMCs from each well were collected and washed with FACS buffer and surface-stained for CD4 (FITC, clone OKT4, Biolegend), CD8 (Brilliant Violet 510™, clone SK1, Biolegend) and CD69 (PE/Cyanine7, clone FN50, Biolegend) according to the manufacturer’s instructions. All data were collected using a flow cytometer (LSRFortessa; BD Biosciences) and analyzed with FlowJo software (Tree Star, Ashland, OR, USA).

### Publicly available HC data

The V(D)J repertoires of T and B cells integrated with 5′ gene expression data from PBMCs of two additional HCs were acquired from the Single Cell Immune Profiling Datasets of 10x genomics, with one donor generated using a 5′ V2 chemistry kit (labeled as HC2), and the other using a 5′ V3 chemistry kit (labeled as HC3) both on Chromium Single Cell Controller (10x genomics). Data could be accessed via https://support.10xgenomics.com/single-cell-vdj/datasets/3.0.0/ for H4 and via https://support.10xgenomics.com/single-cell-vdj/datasets/2.2.0 for H5.

### scRNA-seq and preprocessing

#### Single-cell 5′ RNA sequencing

The library of single-cell 5′ RNA was established following the protocol from 10x genomics Chromium Single Cell Immune Profiling Solution. In brief, the RNA of the captured cells was released by lysis, barcoded via reverse transcription process, and amplified until acquiring sufficient cDNA for constructing 5′ gene expression libraries. The Illumina Novaseq 6000 with PE150 was applied to sequence the 5′ transcripts.

#### Sample demultiplexing

FASTQ files within read2 files were demultiplexed, and the cell barcodes and single-cell 5′ unique molecular identifiers within read1 files were achieved by Cell Ranger Single-Cell Software Suite (v.3.1.0). Each sample was analyzed using Cell Ranger count pipeline with default parameters and GRCh38 as the reference human genome.

### Single-cell transcriptome data analysis

#### Cell aggregation and quality control

Seurat (v.3.1.4)^44^ was applied to aggregate all samples, and perform quality control on raw gene expression matrix obtained from Cell Ranger pipeline. The filtering criteria for each cell were: (1) the number of genes expressed ranges from 200 to 4000, and (2) the percentage of the mitochondrial genes is less than 25%. Cells that did not satisfy the above criteria were discarded. Gene expressions were normalized by log normalization with factor 10,000, and the top 2000 highly variable genes were selected for PCA.

#### Batch-effect correction

Though many genes show significant differences in expression, these differences may come from experimental batch effect. Therefore, Harmony^45^ was utilized for batch-effect correction based on the top 50 principal components of PCA.

#### Cluster identification

After removing the batch effect, dimension reduction was performed using t-SNE algorithm, and cell clusters were identified at resolution 3.0 by FindClusters function of Seurat (v.3.1.4) utilizing a shared nearest neighbor graph constructed by FindNeighbors function. Finally, 59 clusters were obtained.

#### Cell type annotation

The corresponding cell types of the clusters were annotated using SingleR^30^ and Scibet^29^ with the reference datasets of human immune cells. The annotation was corrected manually in accordance with known gene markers.

### Differential gene expression analysis

Differential gene expression analysis was performed using Seurat v.3 (FindMarkers function) with default parameters. We identified differentially expressed genes of each COVID-19 sample by comparing CD4+, CD8+, or B cells of each COVID-19 sample to those cells of all HCs. Genes with adjusted *P* < 0.05 and fold change > 1.5 were considered as significantly upregulated in patients and were used for further GO enrichment analysis.

### GO and pathway enrichment analysis

GO and pathway enrichment analysis were performed using clusterProfiler^46^ (*P*valueCutoff = 0.01 and *q*valueCutoff = 0.05). For each comparison, the top ten enriched pathways containing at least five upregulated genes were kept. Significantly upregulated pathways shared by at least two patients were selected to display in bubble charts.

### Single-cell BCR/TCR V(D)J sequencing and analysis

Chromium single cell V(D)J enriched libraries were quantified, normalized, and sequenced according to the User Guide for Chromium Single Cell V(D)J Reagent kits. The V(D)J enriched libraries were pooled and sequenced with an Illumina NovaSeq 6000 S2 Reagent Kit (300 cycles) (Illumina). BCR/TCR sequences for each single B/T cell were assembled by Cell Ranger vdj pipeline (v.3.1.0), with CDR3 sequences and rearranged full-length BCR/TCR V(D)J segments as well as clonotype frequency obtained. Only productive BCR/TCR sequences were kept for subsequent analysis. The assembled FASTA sequences of IGH chains were collected and assigned germline sequences using Change-O^24^ to perform clonal grouping and mutation analysis. V(D)J segment usage counts were calculated for all cohorts and PCA analysis was performed to compare the V(D)J segment usage among SPs, MPs, and HCs.

### BCR/TCR clonal grouping

All BCR/TCR clonotypes were identified by Cell Ranger vdj pipeline (v.3.1.0). The BCR/TCR clonotypes from SPs, MPs, and HCs were pooled together before carrying out the clonal grouping step. TCR clusters were predicted by GLIPH^27^ to bind the same MHC-restricted peptide antigen. Public TCR clusters were detected for patients and HCs, respectively. BCR clusters were identified using Change-O based on hierarchical clustering approach. First, IGH sequences with the same V and J segment calls and CDR3s of the same length were grouped. Second, IGH sequences within each group were clustered into BCR clusters based on the hamming distances of CDR3 nucleotide sequences. Public BCR clusters of each isotype (IgM, IgA, and IgG) were detected for patients and HCs, separately.

### Clonal abundance and diversity

Clonal abundance distributions were inferred using resampling strategies to correct for variations in sequencing depth.^13^ Clonal diversity distributions were calculated by plotting diversity index^14^ over a range of diversity orders (*q*) to generate a smooth curve. All the corresponding analyses were performed using R package alakazam.^47^

Clonal abundance was defined by the number of BCR or TCR sequences in each cluster that gathered similar clones according to a sequence-based distance measure defined by nucleotide similarity of the junction regions.^24^ The complete clonal abundance distribution were inferred using a novel estimator of the complete clones-rank abundance distribution by separately adjusting the sample relative abundances for the set of clones detected in different donor groups and estimating the relative abundances for the set of clones undetected in the group but inferred to be present in the assemblage. The colored bands indicate the middle 95% percentiles of the clone distribution with 200 bootstrap estimates in each donor group.

The Hill diversity index (^q^D), which measures diversity in a population, was calculated for the set of BCR IGH or TCR clones to determine whether the diversity in the repertoires differed between the donor groups.^47^ For each donor group, the repertoire was subsampled to the number of sequences in the smallest sample and the Hill diversity index was calculated independently in 200 equally spaced *q* values between 0 and 4. Because *q* varies from 0 to infinity, the diversity (^q^D) depends less on rare species and more on common ones as *q* increases, thus encompassing a range of definitions that can be visualized as a single curve. For *q* = 0, the diversity is defined as the total number of clones. As *q* approaches infinity, the diversity is given by one over the frequency of the largest clone. At a given value of *q* (*x* axis), lower values of ^q^D (*y* axis) indicate lower diversity. The colored bands indicate the middle 95% percentiles of the sampled distribution; thus, when three lines are separated and fall outside of the colored band, the difference between the donor groups is significant.

### Mutation and selection analysis

Replacement (R) mutations within subregions of the V segment were identified by comparing the input BCR sequences with the germline sequences using Change-O. In addition, the quantification of selection pressure was performed for SPs, MPs, and HCs using BASELINe^15^ based on an underlying SHM targeting model. The probability density function (PDF) for selection strength in CDR and FWR regions was calculated using a Bayesian estimation model.

### Statistics

DESEQ2^48^ was used to find differential segment usages in BCR and TCR. The *P* value and fold change were plotted as volcano map using ggplot2. Wilcoxon signed ranks test and *t*-test was used pairwise comparisons.

## Supplementary information

Supplementary Materials

## Data Availability

The datasets used for the current study are available to download via http://www.microbiome-bigdata.com/project/SARS-CoV-2/.
