# Correction to: Adaptive immune responses to SARS-CoV-2 infection in severe versus mild individuals

**DOI:** 10.1038/s41392-021-00540-4

**Published:** 2021-04-19

**Authors:** Fan Zhang, Rui Gan, Ziqi Zhen, Xiaoli Hu, Xiang Li, Fengxia Zhou, Ying Liu, Chuangeng Chen, Shuangyu Xie, Bailing Zhang, Xiaoke Wu, Zhiwei Huang

**Affiliations:** 1 HIT Center for Life Sciences, School of Life Science and Technology, Harbin Institute of Technology, Harbin, 150080 China; 2 Department of Infectious Diseases, Heilongjiang Provincial Hospital, Harbin Institute of Technology, Harbin, 150030 China; 3 Harbin Blood Center, Harbin, 150056 China; 4 Centre for Reproductive Medicine, Heilongjiang Provincial Hospital, Harbin Institute of Technology, Harbin, 150030 China; 5 Department of Obstetrics and Gynecology, First Affiliated Hospital, Heilongjiang University of Chinese Medicine, Harbin, 150040 China

Correction to: *Signal Transduction and Targeted Therapy* 10.1038/s41392-020-00263-y, published online 14 August 2020

Since online publication of the article^1^, a lot of researchers worldwide ask for the datasets used in this study. So we revised the “Data availability” section and added the access to our datasets in this correction as following:

Data availability

The datasets used for the current study are available to download via http://www.microbiome-bigdata.com/project/SARS-CoV-2/.

The original article has been corrected.
