# Supplementary material for: Adaptive immune responses to SARS-CoV-2 infection in severe versus mild individuals
Source: Signal Transduct Target Ther. 2020 Aug 14;5:156. doi: 10.1038/s41392-020-00263-y (PMC7426596; doi:10.1038/s41392-020-00263-y)
Supplement: Supplementary file 1 — Supplementary Materials [file 41392_2020_263_MOESM1_ESM.docx]

Supplementary Materials for

Adaptive immune responses to SARS-CoV-2 infection in severe

versus mild individuals

Fan Zhang1, Rui Gan1, Ziqi Zhen1, Xiaoli Hu2, Xiang Li1, Fengxia Zhou1, Ying Liu3, Chuangeng Chen1, Shuangyu Xie1, Bailing Zhang1, Xiaoke Wu4,5 and Zhiwei Huang1*

Correspondence to: huangzhiwei@hit.edu.cn

**This PDF file includes:**

Figures. S1 to S9

Tables S1 to S5

­­


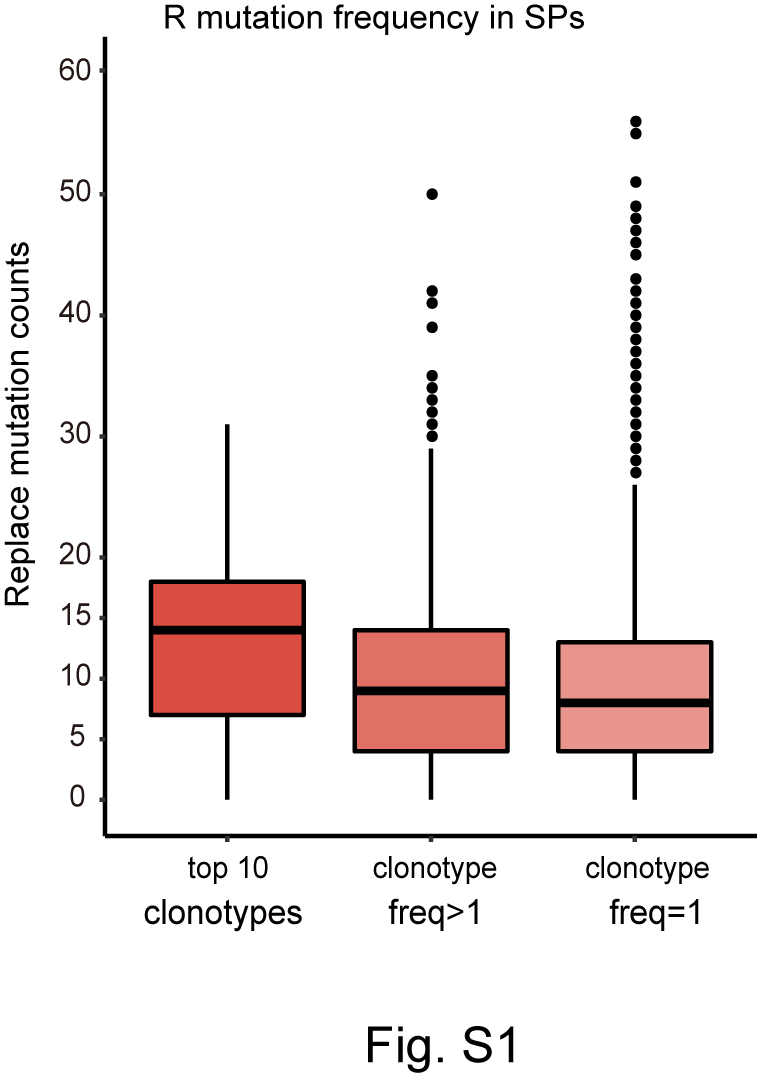


Figure. S1.

Boxplot comparing the distribution of somatic mutation frequencies of IGH V gene segment among differentially expanded clones in severe patients.


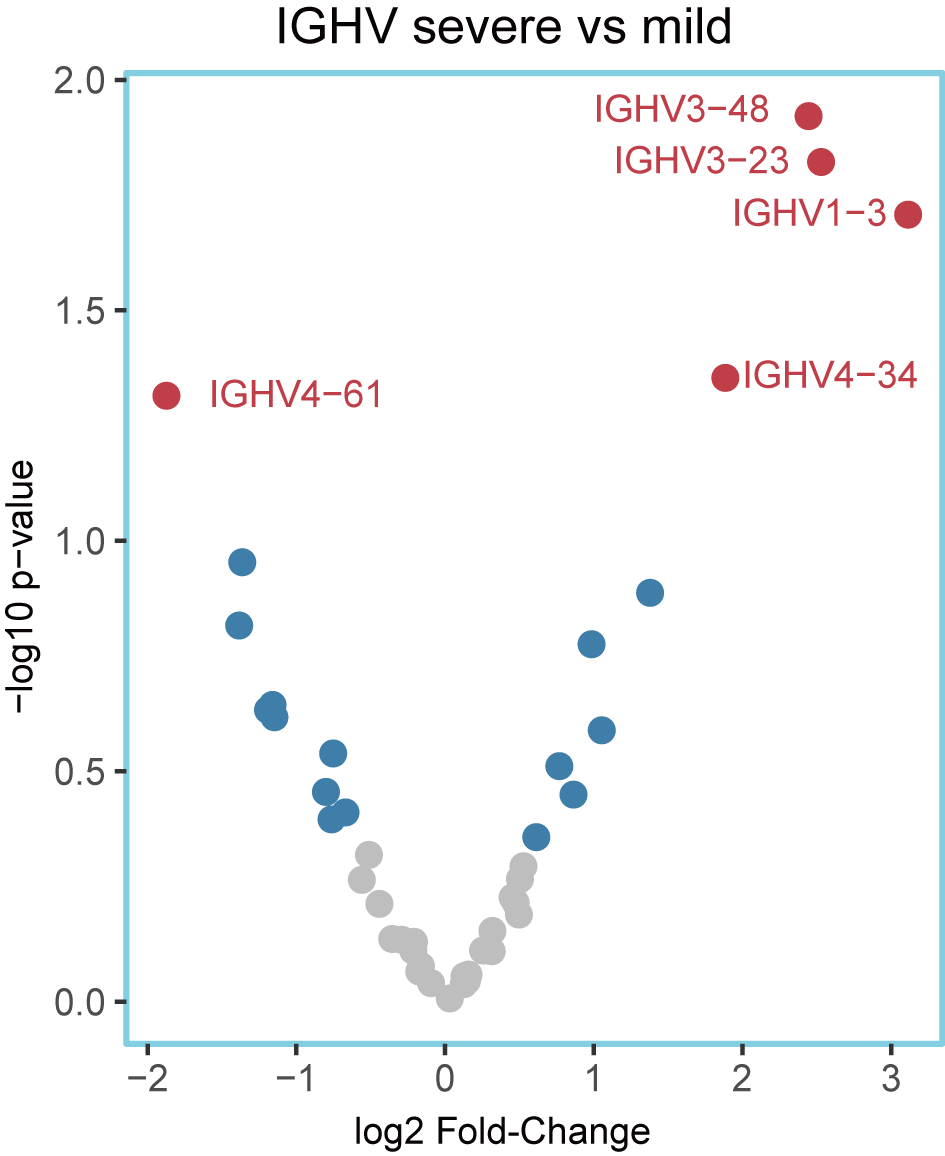


Figure. S2.

Volcano plot representing differences of IGHV gene usage in BCR repertoires. Positive fold-change values denote more frequent IGHV genes in severe COVID-19 patients. Genes with *p-*value < 0.05 are displayed in red. Genes with a *p*-value > 0.05 but fold-change (FC) value > 1.5 are displayed in blue.


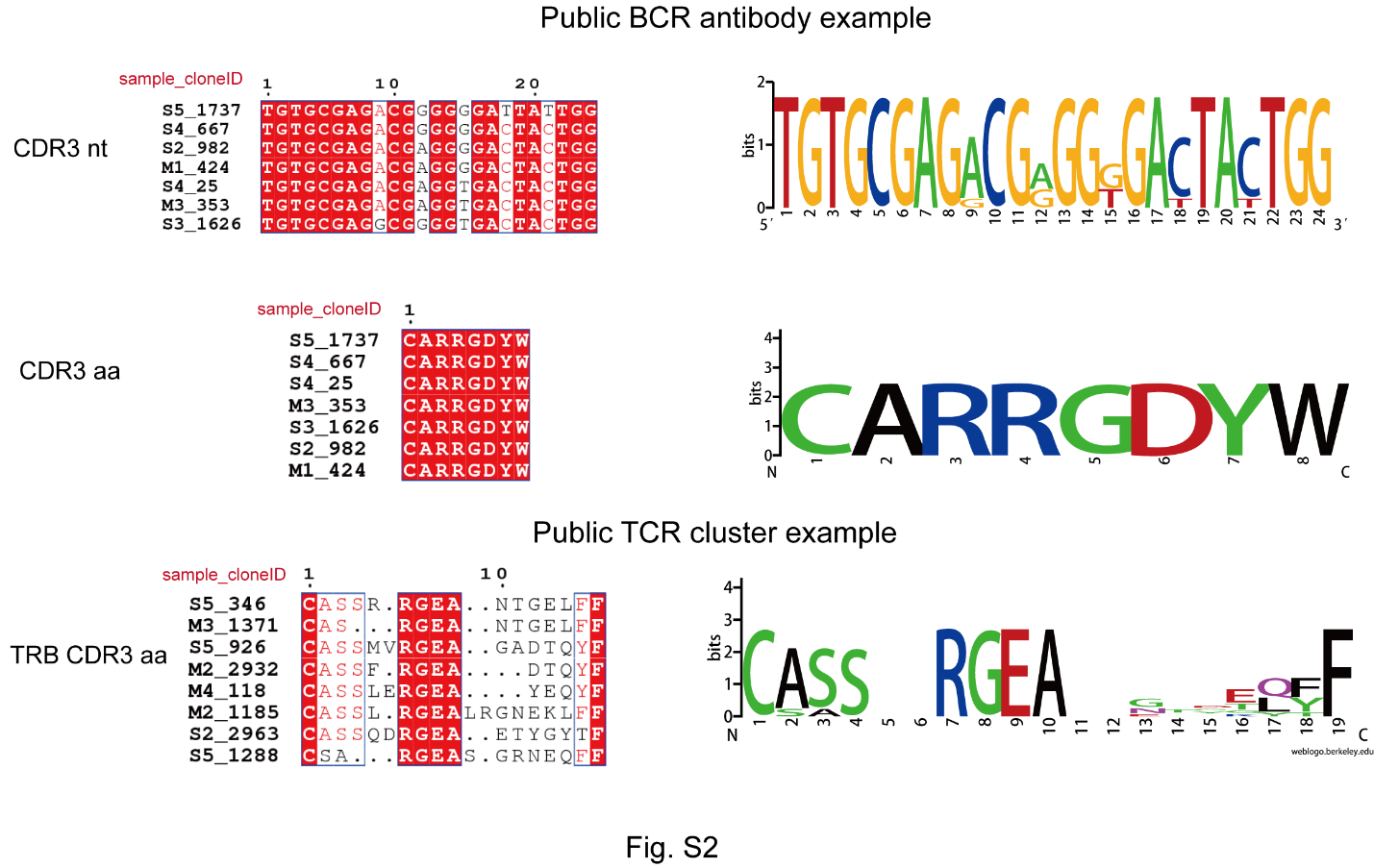


Figure. S3.

Multiple alignment and motif analyses for CDR3 regions of exemplary public BCR cluster and TCR cluster.


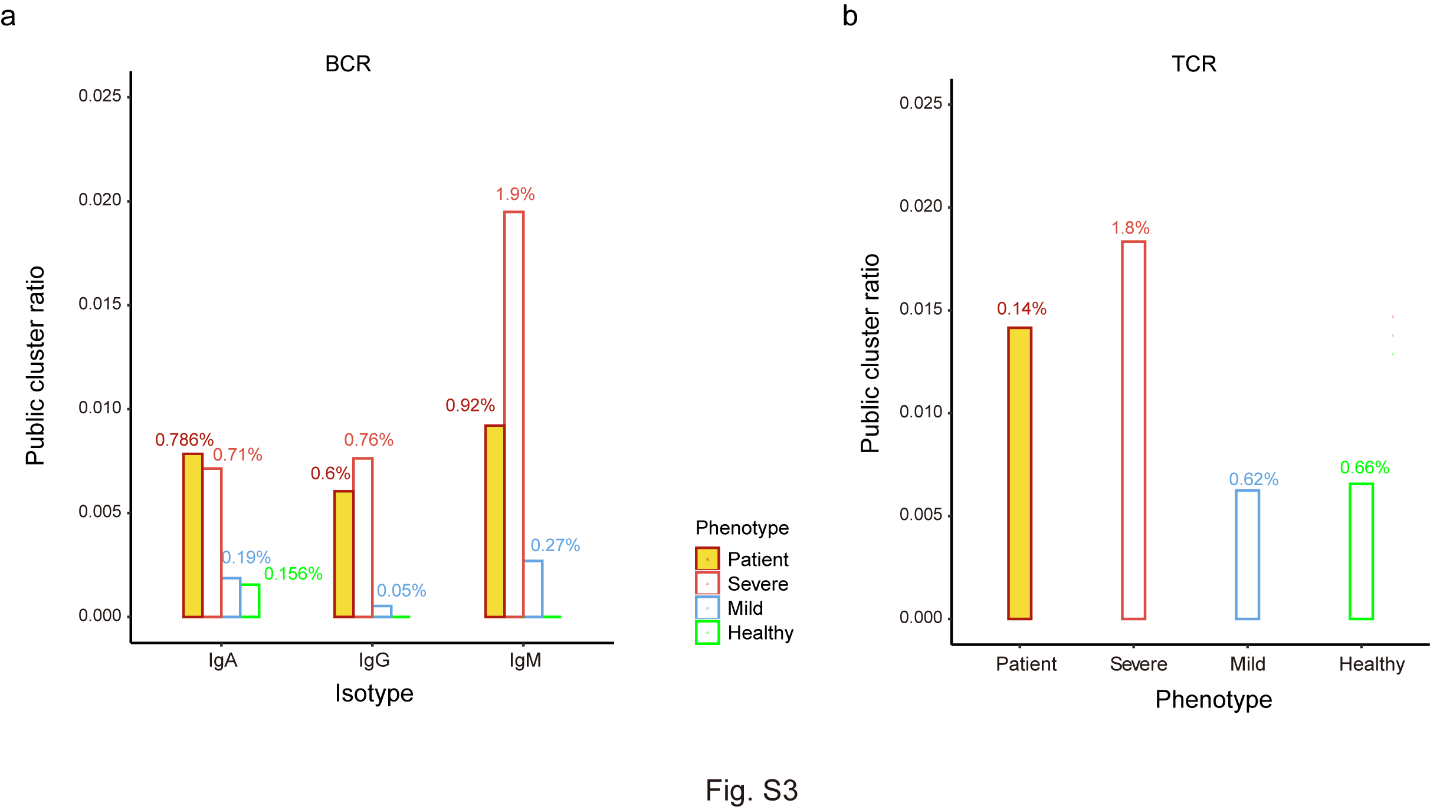


Figure. S4.

Proportions of public isotype (IgA, IgG, IgM) BCR clusters (a) and public TCR clusters (b) shared by severe patients (red), mild patients (blue), healthy controls (green), or at least two COVID-19 patients (orange), respectively.


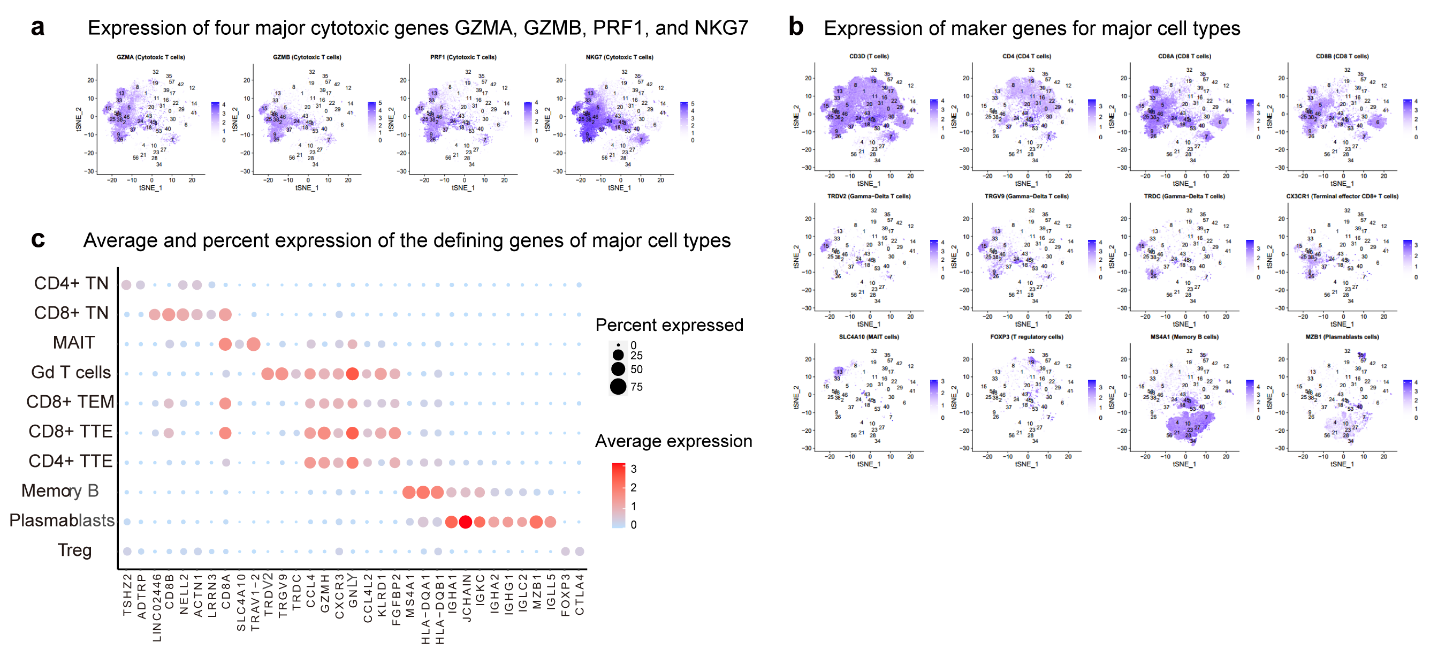


Figure. S5.

**a** Characterization of CTLs based on the expression of four major cytotoxic genes GZMA, GZMB, PRF1, and NKG7. **b** Expression of cell-type specific maker genes for major cell types. For example, Vγ9/Vδ2+ T cells are featured by expressions of TRDV2, TRGV9, and TRDC genes and the t-SNE plot was made for these genes. Similarly, MAIT cells were plotted using the expression of CD3E, CD4, CD8, and SLC4A10 genes. Cell positions are from the t-SNE plot in Fig. 3a. **c** Dot plot showing percentage of cells (dot size) and average expression (red-blue color range) for major cell-type specific genes.


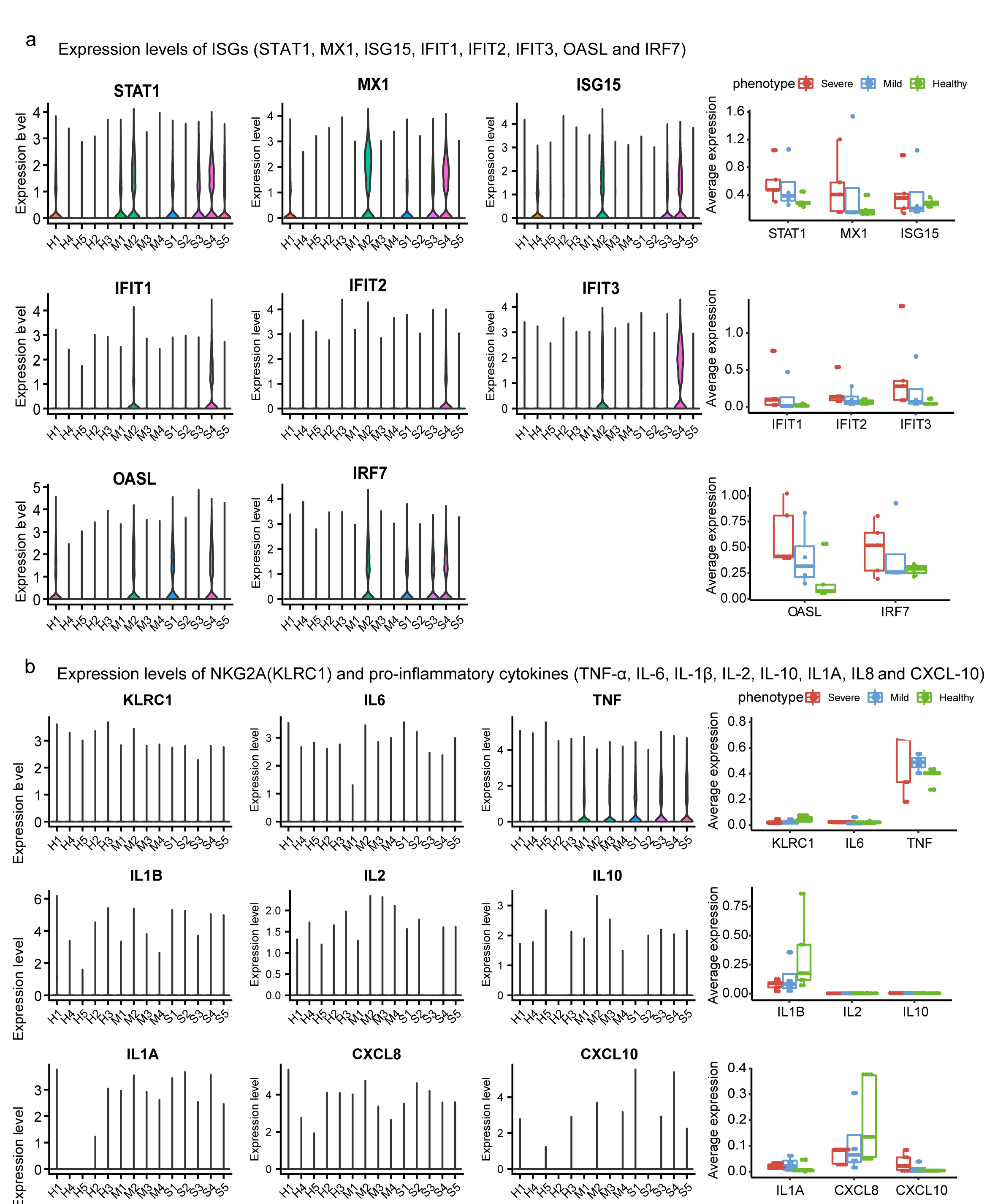


Figure. S6.

**a** Violin plot and boxplot for expression levels of ISGs (STAT1, MX1, ISG15, IFIT1, IFIT2, IFIT3, OASL and IRF7). **b** Violin plot and boxplot for expression levels of NKG2A (KLRC1) and pro-inflammatory cytokines (TNF-α, IL-6, IL-1ß, IL-2, IL-10, IL1A, IL-8, and CXCL-10).


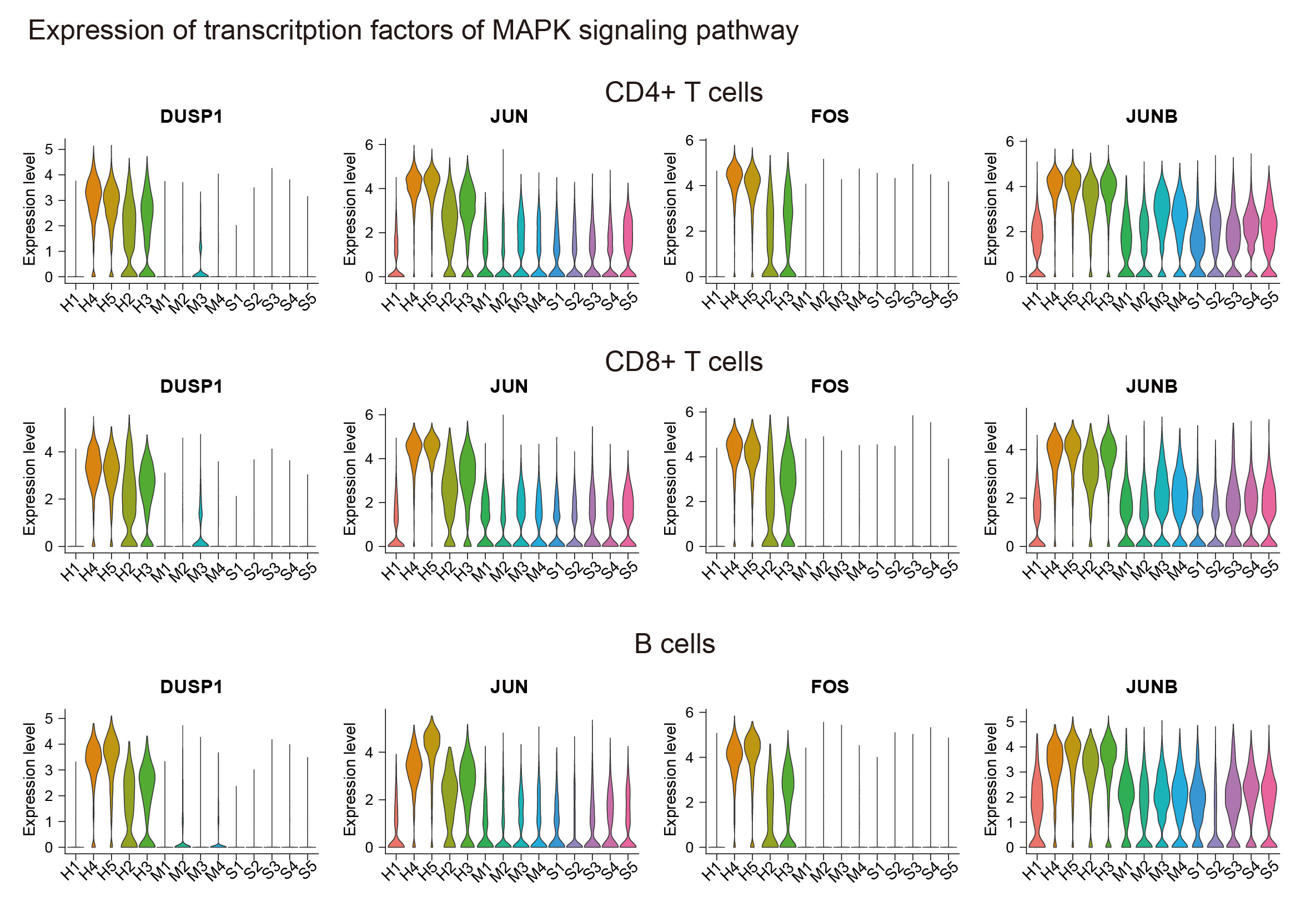


Figure. S7.

Violin plot for expression levels of transcription factors (FOS, JUN, JUNB the DUSP1) of MAPK signaling pathway in CD4+ T cells, CD8+ T cells, and B cells from each donor.


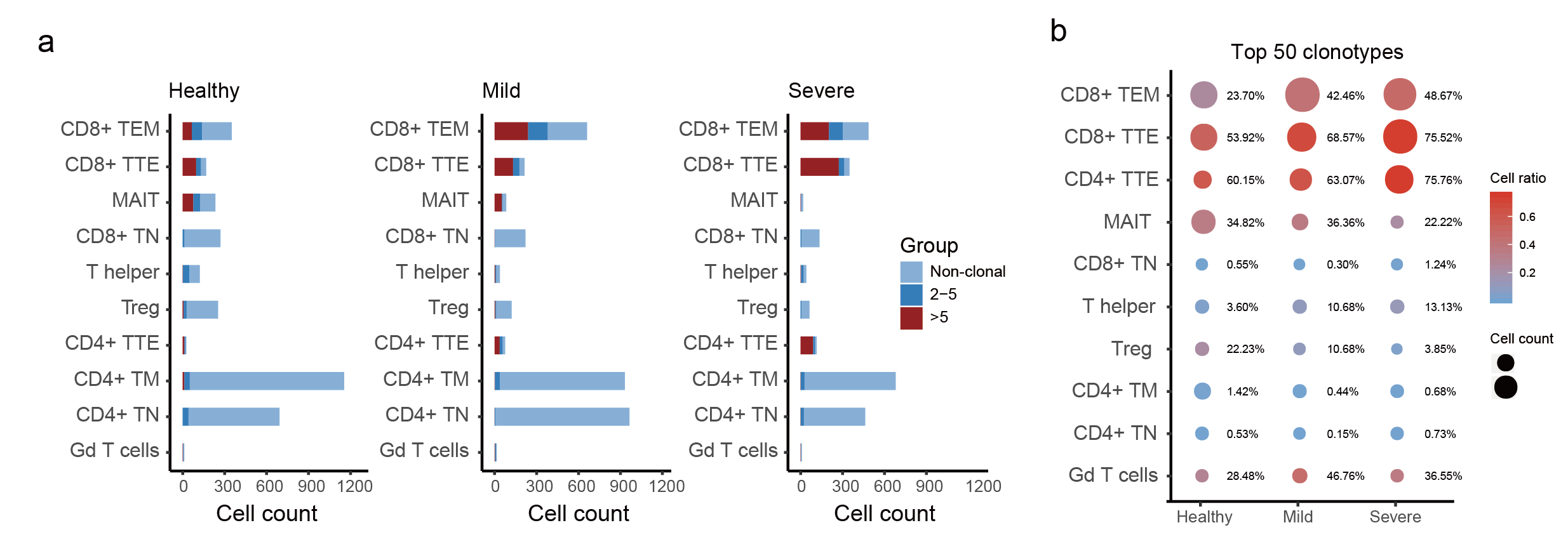


Figure. S8.

T cell subsets exhibited different degrees of clonal expansion in COVID-19 patients. (a) Clonal expansion status of T cell subsets in HCs, MPs, and SPs. Cell counts of highly expanded (frequency>5), moderate expanded (2<frequency<=5), and non-expanded clonotypes are shown as red, blue, and light blue, respectively. (b) Occupancy of the top 50 most expanded TCR clonotypes (y-axis) in each cell cluster (x axis) in HCs, MPs, and SPs.


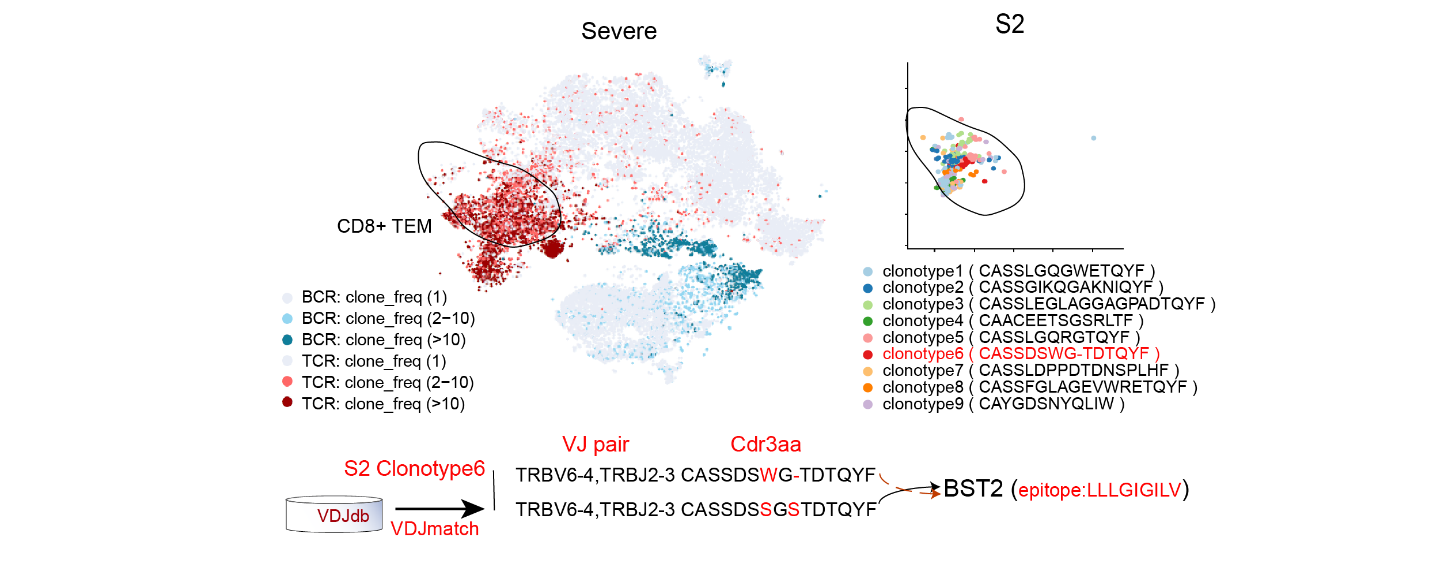


Figure. S9.

The sixth ranked clonotype (frequency=38) in CD8+ TEM cells from S2 was predicted to target a peptide (LLLGIGILV) from human bone marrow stromal cell antigen 2 (BST2).

Table S1.

The demographic and clinical characteristics of nine enrolled COVID-19 convalescent patients.

|  | S1 | S2 | S3 | S4 | S5 | M1 | M2 | M3 | M4 |
| --- | --- | --- | --- | --- | --- | --- | --- | --- | --- |
| Severity | severe | severe | severe | critical | severe | moderate | moderate | mild | mild |
| Age | 67 | 44 | 67 | 50 | 57 | 45 | 56 | 50 | 30 |
| Gender | M | M | F | F | F | F | M | F | M |
| Wuhan exposure history | no | no | no | no | no | no | no | no | yes |
| Symptom onset day | Jan 26, 2020 | Jan 26, 2020 | Jan 23, 2020 | Jan 17, 2020 | Jan 23, 2020 | Jan 23, 2020 | Feb 3, 2020 | Jan 22, 2020 | Jan 15, 2020 |
| First symptom | Fever/Cough | Fever | Fever | Fever | Fever | Fever/Cough | Fever | Fatigue | Fever/Cough |
| Hospitalization date | Feb 9, 2020 | Feb 8, 2020 | Feb 9, 2020 | Jan 23,2020 | Feb 9, 2020 | Feb 9, 2020 | Feb 6, 2020 | none | none |
| Chronic basic disease | hypertension | diabetes | none | none | hypertension | diabetes | Hypertension, diabetes | none | none |
| SARS-CoV-2 | + | + | + | + | + | + | + | + | + |
| Influenza A virus | - | - | - | - | - | - | - | - | - |
| Influenza B virus | - | - | - | - | - | - | - | - | - |
| Respiratory syncytial virus | - | - | - | - | - | - | - | - | - |
| Adenovirus | - | - | - | - | - | - | - | - | - |
| Interfering atomization | Feb 10, 2020 | Feb 8, 2020 | no | Jan 23,2020 | no | no | no | no | no |
| Ribavirin | Feb 10, 2020 | Feb 8, 2020 | Feb 10, 2020 | Jan 23,2020 | no | Feb 10, 2020 | no | no | no |
| Methylprednisolone | yes | yes | no | Yes | no | no | no | no | no |
| BALF sampling date | no | no | no | no | no | no | no | no | no |
| CT finding | Bilaeral pneumonia | Bilaeral pneumonia | Bilaeral pneumonia | Bilaeral pneumonia | Bilaeral pneumonia | Bilaeral pneumonia | Bilaeral pneumonia | no | no |

Table S2.

The number of recovered cells and distinct clonotypes identified for healthy and two distinct severity groups of COVID-19 patients.

| ID | Phenotype | T cell # | B cell # | TClone # | BClone# |
| --- | --- | --- | --- | --- | --- |
| S1 | Severe | 1835 | 1440 | 871 | 963 |
| S2 | Severe | 4115 | 2218 | 2965 | 1006 |
| S3 | Severe | 1417 | 3150 | 1223 | 1852 |
| S4 | Severe | 5182 | 2004 | 4536 | 1178 |
| S5 | Severe | 2801 | 3457 | 1493 | 2367 |
| M1 | Mild | 1286 | 598 | 942 | 586 |
| M2 | Mild | 6007 | 4227 | 4778 | 2798 |
| M3 | Mild | 4510 | 3512 | 3607 | 3237 |
| M4 | Mild | 3243 | 2272 | 2914 | 1967 |
| H1 | Health | 6459 | 1386 | 4999 | 926 |
| H4 | Health | 3009 | 1202 | 2854 | 1196 |
| H5 | Health | 3200 | 1321 | 3016 | 1283 |
| H2 | Health | 2624 | 469 | 1764 | 421 |
| H3 | Health | 4387 | 2643 | 3813 | 1559 |

Table S3.

Public IgG and IgA antibodies.

| cluster | isotype | sample | H_v_gene | H_CDR3 | L_v_gene | L_CDR3 |
| --- | --- | --- | --- | --- | --- | --- |
| 403 | IgG | M3 | IGHV3-64D\|IGHV3-23 | CVNSLRSGWYPVLDYW\|CAKCSYSYGNDAFDIW | IGKV3-20\|IGKV3-20 | CQHYDTIPWTF\|CQQYGSSPRTF |
| 403 | IgG | S4 | IGHV3-23 | CAKCSYSYGNDAFDIW | IGKV3-20 | CQQYGNIPWTF |
| 1641 | IgG | S3 | IGHV1-3 | CARGWDLSSRYYLFDYW | IGKV1-17 | CLQHRDYPISF |
| 1641 | IgG | M2 | IGHV1-3 | CARGWDLSSRYYLFDYW | IGKV1-17 | CLQHRDYPISF |
| 1901 | IgG | M2 | IGHV1-58 | CAATQTCVTMAAGGCLDGLDIW | IGLV3-1 | CQSWDSNTVVF |
| 1901 | IgG | M2 | IGHV3-74\|IGHV1-58 | CARDFGIQDNW\|CAATQTCVTMAAGGCLDGLDIW | IGLV3-1 | CQSWDSNTVVF |
| 1901 | IgG | M3 | IGHV1-58 | CAATQTCVTMAAGGCLDGLDIW | IGLV3-1 | CQSWDSNTVVF |
| 2131 | IgG | S3 | IGHV3-7 | CARFVFGSSSNFFDNW | IGKV2-28 | CMQALHTPWTF |
| 2131 | IgG | S4 | IGHV3-7 | CARFVFGSSSNFFDSW | IGKV2-28 | CMEGLQGPWTF |
| 2963 | IgG | M2 | IGHV4-39 | CARVEGPGTEAFDIW | IGKV3-20 | CQQYGGSRISF |
| 2963 | IgG | S1 | IGHV4-39 | CARVEGPGTEAFDIW | IGKV3-20 | CQQYGGSRISF |
| 3663 | IgG | S3 | IGHV5-51 | CAKRGGLPGTEVQSW | IGKV2-28 | CMQALQIQYTF |
| 3663 | IgG | S4 | IGHV5-51 | CAKRGGIPGTEVQSW | IGKV2-28 | CMQALQIQYTF |
| 5621 | IgG | S5 | IGHV4/OR15-8 | CAREWDYFDYW | IGKV2D-29 | CAQGVDHPYTF |
| 5621 | IgG | M2 | IGHV4/OR15-8 | CAREWDYFDYW | IGKV2D-29 | CAQGVDHPYTF |
| 6041 | IgG | S3 | IGHV3-74 | CVRDNWAPDYW | IGKV2-30 | CMQSTHWPPYTF |
| 6041 | IgG | S4 | IGHV3-74 | CVRDNWSPDYW | IGKV2-30 | CMQGTHWPPYTF |
| 6411 | IgG | S4 | IGHV3-74 | CARDLGYSTSAGWQKHFDYW | IGLV1-40 | CQSYASNLRGVLF |
| 6411 | IgG | S3 | IGHV3-74 | CARDLGYSTSAGWQKYFDYW | IGLV1-40 | CQSFASNVRGVLF |
| 6411 | IgG | S4 | IGHV3-74 | CVRDLGYSTSAGWQKHFDYW | IGLV1-40 | CQSYVSNMRGVLF |
| 8422 | IgG | S3 | IGHV1-2 | CARCLSEYNYGPHELKFDYW | IGKV1-39 | CQQSYNVFTF |
| 8422 | IgG | M2 | IGHV1-2 | CARCLSEYNYGPHELKFDYW | IGKV1-39 | CQQSYNVFTF |
| 8939 | IgG | M2 | IGHV3-21 | CVREDGSSSHYRYAFDYW | IGKV3D-15 | CQQYSDWPPVTF |
| 8939 | IgG | S1 | IGHV3-21 | CVREDGSSSHYRYAFDYW | IGKV3D-15 | CQQYSDWPPVTF |
| 9062 | IgG | S3 | IGHV1-2 | CARGLLGRYSSTCYFEFW | IGKV3-15 | CQENYKWQTF |
| 9062 | IgG | S4 | IGHV1-2 | CARGLLGRYSSTCYFEFW | IGKV3-15 | CQENYKWQTF |
| 10399 | IgG | S5 | IGHV3-23 | CAKDWVDDSAWQSHPLFDYW | IGLV1-44 | CLAWDDSLSGWVF |
| 10399 | IgG | S1 | IGHV3-23 | CAKDWVDDSAWQSHPLFDYW | IGLV1-44 | CLAWDDSLSGWVF |
| 10510 | IgG | S3 | IGHV2-5 | CAHSESCSGSRCYSWTPSGWLDPW | IGKV2D-28 | CLQRPQRPPWTF |
| 10510 | IgG | S4 | IGHV2-5 | CAHSESCSGSRCYSWTPSGWLDPW | IGKV2D-28 | CLQRPQRPPWTF |
| 10821 | IgG | S1 | IGHV4-4 | CARGGGYYFDYW | IGLV8-61 | CVLYVRTGLYVF |
| 10821 | IgG | S2 | IGHV4-4 | CARGGGYYFDYW | IGLV8-61 | CVLYVGYGNYVF |
| 11170 | IgG | S3 | IGHV3-23 | CARSEYCTTGRCQSFDPW | IGKV1-9 | CQQLNRFPITF |
| 11170 | IgG | S4 | IGHV3-23 | CAKSDYCSSGKCQSFDPW | IGKV1-9 | CQQLHRFPITF |
| 11353 | IgG | M2 | IGHV3-74 | CVRDLVGLFDSW | IGKV2D-28 | CMQGLQTPYTF |
| 11353 | IgG | S1 | IGHV3-74 | CVRDLVGLFDSW | IGKV2D-28 | CMQGLQTPYTF |
| 11558 | IgG | S3 | IGHV3-7 | CARSTRSSWNEW | IGLV2-23 | CCSYAGSGTYVF |
| 11558 | IgG | S4 | IGHV3-7 | CARSTRSSWNEW | IGLV2-23 | CCSYAGSGTYVF |
| 11693 | IgG | S3 | IGHV3-23 | CVKRSDALWGHFDYW | IGKV3-15 | CQQYRRWPLTF |
| 11693 | IgG | M2 | IGHV3-23 | CVKRSDALWGHFDYW | IGKV3-15 | CQQYRRWPLTF |
| 264 | IgA | S3 | IGHV4-28 | CARGSFGVVTTPFDSW | IGKV3-20 | CQQYDSSLSLTF |
| 264 | IgA | S5 | IGHV4-28 | CARGSFGVVTTPFDSW | IGKV3-20 | CQQYDSSLSLTF |
| 786 | IgA | S4 | IGHV4-34 | CAAHCSNSNCYHRMDVW | IGLV2-14 | CSSYTTSDTYVF |
| 786 | IgA | S3 | IGHV4-34\|IGHV3-33 | CAAHCSNSNCYHRMDVW\|CATPGNFGYGYFFDYW | IGLV2-14\|IGLV2-14 | CSSYTTSDTYVF\|CSSYTSRSGSILAYVF |
| 1266 | IgA | S4 | IGHV3-15 | CTSDIGPRQDNWFDPW | IGLV7-46 | CFLVHSGDNVIF |
| 1266 | IgA | S3 | IGHV3-15 | CTSDIGPRQDNWFHPW | IGLV7-46 | CFLYYSGDGVIF |
| 2285 | IgA | M2 | IGHV3-49 | CMTLLTVPLIPVW | IGKV1-5 | CQQYNTYWGTF |
| 2285 | IgA | S1 | IGHV3-49 | CMTLLTVPLIPVW | IGKV1-5 | CQQYNTYWGTF |
| 2532 | IgA | S5 | IGHV3-15 | CTTDPEIDSSGTSAYFFWSGFW | IGKV3-20 | CHQYGSSLPLSF |
| 2532 | IgA | M2 | IGHV3-15 | CTTDPEIDSSGTSAYFFWSGFW | IGKV3-20 | CHQYGSSLPLSF |
| 2957 | IgA | M2 | IGHV4-39 | CARFWRGPSYTFDLW | IGKV3-20 | CQHYGSTSWTF |
| 2957 | IgA | S5 | IGHV4-39 | CARFWRGPSYSFDLW | IGKV3-20 | CQHYGSTTWTF |
| 2957 | IgA | S5 | IGHV4-39 | CARFWRGPSYTFDLW | IGKV3-20 | CQHYGSTSWTF |
| 3289 | IgA | S5 | IGHV6-1 | CARDGDLSLSTMDYW | IGLV2-14 | CSSYTRSNTGCVVF |
| 3289 | IgA | S5 | IGHV6-1 | CARDGDLSQSTMDYW | IGLV2-14 | CSSYSSTSVGAVVF |
| 3289 | IgA | M2 | IGHV6-1 | CARDGDLSLSTMDYW | IGLV2-14 | CSSYTRSNTGCVVF |
| 3981 | IgA | M1 | IGHV5-10-1 | CARRPGYDENGYWLYFPYDTW | IGKV1-5 | CQQYNSYPETF |
| 3981 | IgA | S5 | IGHV5-10-1 | CARRPGYDENGYWLYFPYDTW | IGKV1-5 | CQQYNSYPETF |
| 3981 | IgA | M4 | IGHV5-10-1 | CARRPGYDENGYWLYFPYDTW | IGKV1-5 | CQQYNSYPETF |
| 3981 | IgA | M2 | IGHV5-10-1 | CARRPGYDENGYWLYFPYDTW | IGKV1-5 | CQQYNSYPETF |
| 4086 | IgA | S3 | IGHV3-74 | CGKDVHDAAADFR | IGLV1-51 | CASWDASLRAAVF |
| 4086 | IgA | S4 | IGHV3-74 | CGKDVHDAAADFR | IGLV1-51 | CASWDASLRAAVF |
| 4187 | IgA | M2 | IGHV4-4 | CARNEYGPAGVGPFDPW | IGKV3-11 | CQQRSNWPPVTF |
| 4187 | IgA | S5 | IGHV4-4 | CARNEYGPAGVGPFDPW | IGKV3-11 | CQQRSNWPPVTF |
| 4807 | IgA | S3 | IGHV2-5 | CAYRVVGRGLDNW | IGKV1-5 | CQQYNSYPWTF |
| 4807 | IgA | S4 | IGHV2-5 | CAYRVVGRGLDNW | IGKV1-5 | CQQYNSYPWTF |
| 5678 | IgA | S3 | IGHV3-48 | CARGPSSGWALQHW | IGLV2-14 | CAAHRSGATPYVF |
| 5678 | IgA | S4 | IGHV3-48 | CARGPSSGWALQHW | IGLV2-14 | CAAHRSGATPYVF |
| 6365 | IgA | S2 | IGHV1-18 | CAREGFCSGGSCYSGLDDSW | IGLV1-40 | CQSFDSSLSGSKVY |
| 6365 | IgA | S4 | IGHV1-18 | CAREGFCSGGSCYSGLDDSW | IGLV1-40 | CQSFDSSLSGSKVY |
| 6880 | IgA | S4 | IGHV5-51 | CARRLLYDTSGYDWNYFDHW | IGLV1-44 | CAAWDDNLNAYVF |
| 6880 | IgA | S2 | IGHV5-51 | CARRLLYDTSGYDWNYFDHW | IGLV1-44 | CAAWDDNLNAYVF |
| 6989 | IgA | S3 | IGHV1-3 | CARGKSGSPLTTYQYGMDLW | IGLV3-1 | CQAWDTSVVIF |
| 6989 | IgA | S4 | IGHV1-3 | CARGKSGSPLTTYQYGMDVW | IGLV3-1 | CQAWDTNVVVF |
| 7001 | IgA | S4 | IGHV3-21 | CAGGGGPSGSPTVRGGIGPW | IGKV1-5 | CQQYNNYPKTF |
| 7001 | IgA | S3 | IGHV3-21\|IGHV3-23 | CARGGGPSGSTTVRGGIGPW\|CAGVNGDYPYRPWDNW | IGKV1-5\|IGKV3D-15 | CQQYNSYPKTF\|CQQYNNWPLTF |
| 7001 | IgA | S3 | IGHV3-21 | CAGGGGPSGSTTVRGGMGPW | IGKV1-5 | CQQYHTYPKTF |
| 7001 | IgA | S3 | IGHV3-21 | CAGGGGPSGSTTVRGGMGPW | IGKV1-5 | CQQYNSYPKTF |
| 7107 | IgA | S5 | IGHV4-59 | CARGWGGSWYSRYEFQHW | IGLV2-8 | CGSYAGNNHPHVVF |
| 7107 | IgA | M2 | IGHV4-59 | CARGWGGSWYSRYEFQHW | IGLV2-8 | CGSYAGNNHPHVVF |
| 7282 | IgA | S1 | IGHV3-7 | CARSLTGYSEATYW | IGLV7-43 | CLLYYGDSQLWVF |
| 7282 | IgA | M2 | IGHV3-7 | CARSLTGYSEATYW | IGLV7-43 | CLLYYGDSQLWVF |
| 8094 | IgA | S3 | IGHV1-46 | CARDNSISGLTGGLIAAWWFDPW | IGLV3-19 | CNSRDNSRGVFWLF |
| 8094 | IgA | S4 | IGHV1-46 | CARDNSISGLTGGLIAAWWFDPW | IGLV3-19 | CNSRDSSGNVFWLF |
| 8460 | IgA | S5 | IGHV3-33 | CATAGYCSGDCYASGMDVW | IGKV3-11 | CQQRRNWPPVYTF |
| 8460 | IgA | M2 | IGHV3-33 | CATAGYCSGDCYASGMDVW | IGKV3-11 | CQQRRNWPPVYTF |
| 8560 | IgA | S3 | IGHV4-59 | CVREINVVEPAARYFDYW | IGKV1D-39 | CQQTYSTPRTF |
| 8560 | IgA | M2 | IGHV4-59 | CVREINVVEPAARYFDYW | IGKV1D-39 | CQQTYSTPRTF |
| 9330 | IgA | M2 | IGHV1-2 | CARGAGFCSITSCYQLDPW | IGLV3-19 | CNSRDSLRDHWVF |
| 9330 | IgA | S1 | IGHV1-2 | CARGAGFCSITSCYELDPW | IGLV3-19 | CNSRDSLSNHWVF |
| 9330 | IgA | S1 | IGHV1-2 | CARGAGFCSITSCYQLDPW | IGLV3-19 | CNSRDSLRDHWVF |
| 9330 | IgA | S1 | IGHV1-2 | CARGAGFCSITSCYELDPW | IGLV3-19 | CASRDSRSSHWFF |
| 9330 | IgA | S1 | IGHV1-2 | CARGAGFCSITSCYELDPW | IGLV3-19 | CASRDSRSSHWFF |
| 9330 | IgA | M2 | IGHV1-2 | CARGAGFCSITSCYELDPW | IGLV3-19 | CNSRDSLSNHWVF |
| 9330 | IgA | S5 | IGHV1-2 | CARGAGFCSITSCYQLDPW | IGLV3-19 | CNSRDSLRDHWVF |
| 10123 | IgA | M2 | IGHV3-33 | CTRDRARAFDIW | IGKV2D-29 | CMQAVQLPRTF |
| 10123 | IgA | S5 | IGHV3-33 | CTRDRARAFDIW | IGKV2D-29 | CMQAVQLPRTF |
| 10747 | IgA | M2 | IGHV3-33 | CAKATGNWDYASSFGHW | IGKV3-15 | CQQYYNWPSWTF |
| 10747 | IgA | S1 | IGHV3-33 | CAKATGNWDYASSFGHW | IGKV3-15 | CQQYYNWPSWTF |
| 11165 | IgA | S3 | IGHV3-23 | CAKVMLQGVVITSTFDYW | IGKV1-33 | CQQYDRLPLTF |
| 11165 | IgA | S4 | IGHV3-23 | CAKVMLQGVVITSTFDYW | IGKV1-33 | CQHYDSLPLTF |

Table S4.

List of marker genes used for T and B cell subsets annotation.

| Subtype | Description | Markers |
| --- | --- | --- |
| CD4+ TM | CD4+ memory T cells | CD3E, CD4, IL7R, ANXA1, S1PR1 |
| CD4+ TN | CD4+ naive T cells | CD3E, CD4, CCR7, LEF1, TCF7, CD27, CD28, SELL, S1PR1 |
| CD4+ TRAEM/TEFF | CD4+ recently activated effector memory or effector T cells (CTL) | CD3E, CD4, KLRG1, CX3CR1, NKG7, PRF1, GNLY, GZMH, TBX21, CTSW, S1PR1, S1PR5 |
| Treg | T regulatory cells | CD3E, CD4, FOXP3, IL2RA, IL10RA, IKZF2, PTKN2, CDC25B, S1PR4 |
| T helper | T helper cells | CD3E, CD4, IL6ST |
| CD8+ TN | CD8+ naive T cells | CD3E, CD4, CD8A, CD8B, CCR7, LEF1, TCF7, CD27, CD28, SELL, S1PR1 |
| CD8+ MAIT | CD8+ mucosal-associated invariant T cells | CD3E, CD4, CD8A, CD8B, SLC4A10, KLRB1, ZBTB16, NCR3, RORC, RORA |
| CD8+ TEM | CD8+ effector memory T cells | CD3E, CD4, CD8A, CD8B, GZMK, CXCR4, CXCR3, CD44 |
| CD8+ TRAEM/TEFF | CD8+ recently activated effector memory or effector T cells (CTL) | CD3E, CD4, CD8A, CD8B, KLRG1, CX3CR1, FCGR3A, FGFBP2, PRF1, GZMH, TBX21, EOMES, S1PR1, S1PR5 |
| Delta-Gamma T Cells | Delta-Gamma T cells | TRDV2, TRGV9, TRDC |
| Memory B | Memory B cell | CD27, CD38, IGHG1, IGHA1 |
| Plasmablasts | Plasmablasts | XBP1，MZB1, IGHA, IGHG, CD38, loss of MS4A1(CD20) |

Table S5.

Peptides used for ELISPOT.

| peptide id | peptide sequence | source protein | pool |
| --- | --- | --- | --- |
| p001 | FVSEETGTL | E | Pool 2 |
| p002 | YVYSRVKNL | E | Pool 2 |
| p003 | SEETGTLIV | E | Pool 2 |
| p004 | KPSFYVYSRVKNLNS | E | Pool 2 |
| p005 | YVYSRVKNLNSS | E | Pool 2 |
| p006 | VKPSFYVYSRVKNLN | E | Pool 2 |
| p007 | ATSRTLSYY | M | Pool 3 |
| p008 | FAYANRNRF | M | Pool 3 |
| p009 | RIAGHHLGR | M | Pool 3 |
| p010 | RLFARTRSM | M | Pool 3 |
| p011 | SYFIASFRL | M | Pool 3 |
| p012 | YFIASFRLF | M | Pool 3 |
| p013 | GLMWLSYFI | M | Pool 3 |
| p014 | IGNYKLNTDHSSSSD | M | Pool 3 |
| p015 | SYYKLGASQRVA | M | Pool 3 |
| p016 | RLFARTRSMWSF | M | Pool 3 |
| p017 | IASFRLFARTRSMWS | M | Pool 3 |
| p018 | FVLAAVYRI | M | Pool 3 |
| p019 | FAPSASAFF | N | Pool 4 |
| p021 | KAYNVTQAF | N | Pool 4 |
| p022 | KTFPPTEPK | N | Pool 4 |
| p022 | LQLPQGTTL | N | Pool 4 |
| p023 | ATKAYNVTQAFGRRG | N | Pool 4 |
| p024 | KLDDKDPNF | N | Pool 4 |
| p025 | AALTNNVAF | NSP12 | Pool 8 |
| p026 | RTAPHGHVM | NSP1 | Pool 10 |
| p027 | IDSYFVVKRHTFSNY | NSP12 | Pool 8 |
| p028 | YLASGGQPI | NSP10 | Pool 10 |
| p029 | STVLSFCAF | NSP10 | Pool 10 |
| p030 | GKYVQIPTTCAN | NSP10 | Pool 10 |
| p031 | LKGKYVQIPTTCAND | NSP10 | Pool 10 |
| p032 | GKYVQIPTTCANDPV | NSP10 | Pool 10 |
| p033 | VKNYFITDAQTGSSK | NSP15 | Pool 6 |
| p034 | QSFLNGFAV | NSP11 | Pool 10 |
| p035 | ALGGSVAIK | NSP16 | Pool 6 |
| p036 | AMYTPHTVL | NSP12 | Pool 8 |
| p037 | AYANSVFNI | NSP12 | Pool 8 |
| p038 | DTDFVNEFY | NSP12 | Pool 8 |
| P039 | FAYTKRNVI | NSP12 | Pool 8 |
| p040 | FVNEFYAYL | NSP12 | Pool 8 |
| p041 | FYGGWHNML | NSP12 | Pool 8 |
| p042 | HLMGWDYPK | NSP12 | Pool 8 |
| p043 | HLYLQYIRK | NSP12 | Pool 8 |
| p044 | IMASLVLAR | NSP12 | Pool 8 |
| p045 | KSAGFPFNK | NSP12 | Pool 8 |
| p046 | LLADKFPVL | NSP14 | Pool 7 |
| p047 | LMIERFVSL | NSP12 | Pool 8 |
| p048 | LTNDNTSRY | NSP12 | Pool 8 |
| p049 | LVASIKNFK | NSP12 | Pool 8 |
| p050 | MTNRQFHQK | NSP12 | Pool 8 |
| p051 | NLIDSYFVV | NSP12 | Pool 8 |
| p052 | NLLKDCPAV | NSP12 | Pool 8 |
| p053 | NVIPTITQM | NSP12 | Pool 8 |
| p054 | QEYADVFHL | NSP12 | Pool 8 |
| p055 | RAMPNMLRI | NSP12 | Pool 8 |
| p056 | RQFHQKLLK | NSP12 | Pool 8 |
| p057 | RYKLEGYAF | NSP15 | Pool 6 |
| p058 | STVFPPTSF | NSP12 | Pool 8 |
| p059 | TMADLVYAL | NSP12 | Pool 8 |
| p060 | VVYRGTTTY | NSP13 | Pool 7 |
| p061 | YADVFHLYL | NSP12 | Pool 8 |
| p062 | YLDAYNMMI | NSP14 | Pool 7 |
| p063 | YTMADLVYA | NSP12 | Pool 8 |
| p064 | TQMNLKYAI | NSP12 | Pool 8 |
| p065 | FFFAQDGNAAIS | NSP12 | Pool 8 |
| p066 | GLVASIKNFKSV | NSP12 | Pool 8 |
| p067 | HRFYRLANECAQ | NSP12 | Pool 8 |
| p068 | AQCFKMFYK | NSP13 | Pool 7 |
| p069 | AQLPAPRTL | NSP13 | Pool 7 |
| p070 | KLFAAETLK | NSP13 | Pool 7 |
| p071 | KLSYGIATV | NSP13 | Pool 7 |
| p072 | KSHKPPISF | NSP13 | Pool 7 |
| p073 | KTIGPDMFL | NSP13 | Pool 7 |
| p074 | QAWQPGVAM | NSP16 | Pool 6 |
| p075 | RELHLSWEV | NSP13 | Pool 7 |
| p076 | REQIDGYVM | NSP16 | Pool 6 |
| p077 | VTDVTQLYL | NSP13 | Pool 7 |
| p078 | PETYFTQSRNLQEFK | NSP15 | Pool 6 |
| p079 | RAKHYVYIGDPAQLP | NSP13 | Pool 7 |
| p080 | SPYNSQNAVASK | NSP13 | Pool 7 |
| p081 | LKLFAAETLKAT | NSP13 | Pool 7 |
| p082 | HANEYRLYL | NSP14 | Pool 7 |
| p083 | KRVDWTIEY | NSP14 | Pool 7 |
| p084 | MMISAGFSL | NSP14 | Pool 7 |
| p085 | MTYRRLISM | NSP14 | Pool 7 |
| p086 | RLISMMGFK | NSP14 | Pool 7 |
| p087 | SLYVNKHAF | NSP14 | Pool 7 |
| p088 | SYATHSDKF | NSP14 | Pool 7 |
| p089 | TYACWHHSI | NSP14 | Pool 7 |
| p090 | YQVNGYPNM | NSP14 | Pool 7 |
| p091 | HVASCDAIM | NSP14 | Pool 7 |
| p092 | TYRRLISMMGFK | NSP14 | Pool 7 |
| p093 | FELEDFIPM | NSP15 | Pool 6 |
| p094 | FENKTTLPV | NSP15 | Pool 6 |
| p095 | IINNTVYTK | NSP15 | Pool 6 |
| p096 | KVDGVDVEL | NSP15 | Pool 6 |
| p097 | KVDGVVQQL | NSP15 | Pool 6 |
| p098 | TTLPVNVAF | NSP15 | Pool 6 |
| p099 | KNYFITDAQTGSSKC | NSP15 | Pool 6 |
| p100 | FVSDADSTL | NSP16 | Pool 6 |
| p101 | GVAMPNLYK | NSP16 | Pool 6 |
| p102 | GVAPGTAVL | NSP16 | Pool 6 |
| p103 | KYTQLCQYL | NSP16 | Pool 6 |
| p104 | TEHSWNADL | NSP16 | Pool 6 |
| p105 | VAMPNLYKM | NSP16 | Pool 6 |
| p106 | YVMHANYIF | NSP16 | Pool 6 |
| p107 | KGRLIIRENNRVVIS | NSP16 | Pool 6 |
| p108 | CQYLNTLTLAVP | NSP16 | Pool 6 |
| p109 | TAFVTNVNASSS | NSP16 | Pool 6 |
| p110 | IQLSSYSLF | NSP16 | Pool 6 |
| p111 | GETLPTEVL | NSP2 | Pool 10 |
| p112 | SEVGPEHSL | NSP2 | Pool 10 |
| p113 | VTNNTFTLK | NSP2 | Pool 10 |
| p114 | YELQTPFEI | NSP2 | Pool 10 |
| p115 | SGVTFQSAVKRTIKG | NSP5/6 | Pool 10 |
| p116 | AEWFLAYIL | NSP3C | Pool 9 |
| p117 | GEAANFCAL | NSP3C | Pool 9 |
| p118 | HEGKTFYVL | NSP3C | Pool 9 |
| p119 | ILKPANNSL | NSP3C | Pool 9 |
| p120 | NYMPYFFTL | NSP3C | Pool 9 |
| p121 | SLDNVLSTF | NSP3C | Pool 9 |
| p122 | STFNVPMEK | NSP3C | Pool 9 |
| p123 | TTIKPVTYK | NSP3C | Pool 9 |
| p124 | TYKPNTWCI | NSP3C | Pool 9 |
| p125 | VMYMGTLSY | NSP3C | Pool 9 |
| p126 | VQMAPISAM | NSP3C | Pool 9 |
| p127 | YAKPFLNKV | NSP3C | Pool 9 |
| p128 | YLNSTNVTI | NSP3C | Pool 9 |
| p129 | YVDNSSLTI | NSP3C | Pool 9 |
| p130 | YVLPNDDTL | NSP3C | Pool 9 |
| p131 | YVNTFSSTF | NSP3C | Pool 9 |
| p132 | YYHTTDPSF | NSP3C | Pool 9 |
| p133 | RTIKVFTTV | NSP3C | Pool 9 |
| p134 | SHFVNLDNLRAN | NSP3C | Pool 9 |
| p135 | EVRTIKVFTTVDNIN | NSP3C | Pool 9 |
| p136 | IINLVQMAPISAMVR | NSP3C | Pool 9 |
| p137 | FGADPIHSL | NSP3N | Pool 9 |
| p138 | FLARGIVFM | NSP6 | Pool 10 |
| p139 | KTLQPVSEL | NSP3N | Pool 9 |
| p140 | LLSAGIFGA | NSP3N | Pool 9 |
| p141 | LVSDIDITF | NSP3N | Pool 9 |
| p142 | PTDNYITTY | NSP3N | Pool 9 |
| p143 | VLSGHNLAK | NSP3N | Pool 9 |
| p144 | VVNAANVYL | NSP3N | Pool 9 |
| p145 | WSMATYYLF | NSP3N | Pool 9 |
| p146 | YLITPVHVM | NSP4 | Pool 7 |
| p147 | KPLEFGATSAALQPE | NSP3N | Pool 9 |
| p148 | VKPFITESKPSVEQR | NSP3N | Pool 9 |
| p149 | ALCTFLLNK | NSP4 | Pool 7 |
| p150 | FLAHIQWMV | NSP4 | Pool 7 |
| p151 | FLLNKEMYL | NSP4 | Pool 7 |
| p152 | FSAVGNICY | NSP4 | Pool 7 |
| p153 | FYLTNDVSF | NSP4 | Pool 7 |
| p154 | GEYSHVVAF | NSP4 | Pool 7 |
| p155 | IYLYLTFYL | NSP4 | Pool 7 |
| p156 | LLNKEMYLK | NSP4 | Pool 7 |
| p157 | LMDGSIIQF | NSP4 | Pool 7 |
| p158 | TQYNRYLAL | NSP4 | Pool 7 |
| p159 | VAAIFYLITPVHVMS | NSP4 | Pool 7 |
| p160 | FLNRFTTTL | NSP5 | Pool 10 |
| p161 | GTDLEGNFY | NSP5 | Pool 10 |
| p162 | QTFSVLACY | NSP5 | Pool 10 |
| p163 | FLLPSLATV | NSP6 | Pool 10 |
| p164 | KLKDCVMYA | NSP6 | Pool 10 |
| p165 | SAFAMMFVK | NSP6 | Pool 10 |
| p166 | VYMPASWVM | NSP6 | Pool 10 |
| p167 | EAFEKMVSL | NSP7 | Pool 10 |
| p168 | EAFEKMVSLLSV | NSP7 | Pool 10 |
| p169 | MADQAMTQM | NSP8 | Pool 10 |
| p170 | SEFDRDAAM | NSP8 | Pool 10 |
| p171 | SSLPSYAAF | NSP8 | Pool 10 |
| p172 | TMLFTMLRK | NSP8 | Pool 10 |
| p173 | SEFSSLPSYAAF | NSP8 | Pool 10 |
| p174 | ALAYYNTTK | NSP9 | Pool 10 |
| p175 | CTDDNALAY | NSP9 | Pool 10 |
| p176 | KVKYLYFIK | NSP9 | Pool 10 |
| p177 | LSDLQDLKW | NSP9 | Pool 10 |
| p178 | NVFAFPFTI | ORF10 | Pool 11 |
| p179 | MGYINVFAF | ORF10 | Pool 11 |
| p180 | ITFDNLKTL | ORF1ab_other | Pool 9 |
| p181 | HLDGEVITF | ORF1ab_other | Pool 9 |
| p182 | DGEVITFDNLKTLLS | ORF1ab_other | Pool 9 |
| p183 | NPTTFHLDGEVITFD | ORF1ab_other | Pool 9 |
| p184 | ALSKGVHFV | ORF3 | Pool 11 |
| p185 | ATIPIQASL | ORF3 | Pool 11 |
| p186 | FTSDYYQLY | ORF3 | Pool 11 |
| p187 | LLYDANYFL | ORF3 | Pool 11 |
| p188 | VYFLQSINF | ORF3 | Pool 11 |
| p189 | YYQLYSTQL | ORF3 | Pool 11 |
| p190 | VRIIMRLWL | ORF3 | Pool 11 |
| p191 | HTIDGSSGV | ORF3 | Pool 11 |
| p192 | HLVDFQVTI | ORF6 | Pool 11 |
| p193 | GTYEGNSPF | ORF7a | Pool 11 |
| p194 | QELYSPIFL | ORF7a | Pool 11 |
| p195 | VKHVYQLRARSVSPK | ORF7a | Pool 11 |
| p196 | IIFWFSLEL | ORF7b | Pool 11 |
| p197 | FLAFLLFLV | ORF7b | Pool 11 |
| p198 | SFYEDFLEY | ORF8 | Pool 11 |
| p199 | YIDIGNYTV | ORF8 | Pool 11 |
| p200 | GIITTVAAF | ORF8 | Pool 11 |
| p201 | AALQIPFAM | S | Pool 5 |
| p202 | AEIRASANL | S | Pool 5 |
| p203 | FEYVSQPFL | S | Pool 5 |
| p203m | FQFCNDPFL | S | Pool 5 |
| p204 | IAIPTNFTI | S | Pool 5 |
| p205 | LQIPFAMQM | S | Pool 5 |
| p206 | LTDEMIAQY | S | Pool 5 |
| p207 | MIAQYTSAL | S | Pool 5 |
| p208 | QSAPHGVVF | S | Pool 5 |
| p209 | SIIAYTMSL | S | Pool 5 |
| p210 | VLNDILSRL | S | Pool 5 |
| p211 | YLQPRTFLL | S | Pool 5 |
| p212 | FVFLVLLPL | S | Pool 5 |
| p212m | FVFFVLLPL | S | Pool 5 |
| p213 | FTISVTTEI | S | Pool 5 |
| p214 | VGYLQPRTF | S | Pool 5 |
| p215 | VVFLHVTYV | S | Pool 5 |
| p216 | FQTLLALHRSYLTPG | S | Pool 5 |
| p216m1 | FQTLLALHRRYLTPG | S | Pool 5 |
| p216m2 | FQILLALHRSYLTPG | S | Pool 5 |
| p217 | GRDIADTTDAVRDPQ | S | Pool 5 |
| p218 | TPTWRVYSTGSNVFQ | S | Pool 5 |
| p219 | GVYYPDKVFRSS | S | Pool 5 |
| p220 | TLVKQLSSNFGA | S | Pool 5 |
| p221 | SQSIIAYTMSLGAEN | S | Pool 5 |
| p222 | IPTNFTISVTTEILP | S | Pool 5 |
| p223 | AAAYYVGYLQPRTFL | S | Pool 5 |
| p224 | APHGVVFLHVTYVPA | S | Pool 5 |
| p225 | RLDKVEAEV | S | Pool 5 |
| p225m | RLDPPEAEV | S | Pool 5 |
| p226 | GVYFASTEK | S | Pool 5 |
| p226m | GVYFASTDK | S | Pool 5 |
| p227 | TLKSFTVEK | S | Pool 5 |
| p228 | QIYKTPPIK | S | Pool 5 |
| p229 | MTSCCSCLK | S | Pool 5 |
| p230 | GVYYHKNNK | S | Pool 5 |
| p231 | LEILDITPC | S | Pool 5 |
| p232 | KIADYNYKL | S-RBD | Pool 5 |
| p232m | VIADYNYKL | S-RBD | Pool 5 |
| p233 | NSASFSTFK | S-RBD | Pool 5 |
| p234 | NYNYLYRLF | S-RBD | Pool 5 |
| p235 | RLFRKSNLK | S-RBD | Pool 5 |
| p236 | VTYVPAQEK | S | Pool 5 |
| p237 | VYSTGSNVF | S | Pool 5 |
| p238 | YGFQPTNGV | S-RBD | Pool 5 |
| p239 | SYGFQPTNGVGYQPY | S-RBD | Pool 5 |
| p240 | GEVFNATRF | S-RBD | Pool 5 |
| p240m | GEVFNATKF | S-RBD | Pool 5 |
| p241 | NVYADSFVI | S-RBD | Pool 5 |
| p241m | NVYADSFVV | S-RBD | Pool 5 |
| p242 | IPFAMQMAY | S | Pool 5 |
| p243 | FIAGLIAIV | S | Pool 5 |
| p244 | YSSANNCTF | S | Pool 5 |
| p245 | TRFQTLLAL | S | Pool 5 |
| p245m | TRFQILLAL | S | Pool 5 |
| p246 | ITDAVDCAL | S | Pool 5 |
| p247 | KKFLPFQQF | S | Pool 5 |
| p248 | HADQLTPTW | S | Pool 5 |
| p249 | VASQSIIAY | S | Pool 5 |
| p250 | NLNESLIDL | S | Pool 5 |
| p251 | VVNQNAQAL | S | Pool 5 |
| p252 | FAMQMAYRF | S | Pool 5 |
| p253 | IANQFNSAI | S | Pool 5 |
| p253m | IANQFNSVI | S | Pool 5 |
| p254 | VYDPLQPEL | S | Pool 5 |
| p255 | RLNEVAKNL | S | Pool 5 |
| p256 | VAKNLNESL | S | Pool 5 |
| p257 | QYIKWPWYI | S | Pool 5 |
| p258 | IAIVMVTIM | S | Pool 5 |
| p259 | VRFPNITNL | S-RBD | Pool 5 |
| p260 | GTHWFVTQR | S | Pool 5 |
| p261 | SVLYNSASF | S-RBD | Pool 5 |
| p262 | SSTASALGK | S | Pool 5 |
| p263 | NQKLIANQF | S | Pool 5 |
| p264 | KEIDRLNEV | S | Pool 5 |
| p265 | YQPYRVVVL | S-RBD | Pool 5 |
|  |  |  |  |
